# *In vivo* implementation of a synthetic metabolic pathway for the carbon-conserving conversion of glycolaldehyde to acetyl-CoA

**DOI:** 10.3389/fbioe.2023.1125544

**Published:** 2023-02-09

**Authors:** Nils Wagner, Frederik Bade, Elly Straube, Kenny Rabe, Cláudio J. R. Frazão, Thomas Walther

**Affiliations:** TU Dresden, Institute of Natural Materials Technology, Dresden, Germany

**Keywords:** synthetic metabolic pathway, ethylene glycol, glycolaldehyde, acetyl-CoA, arabinose 5-phosphate, *Escherichia coli*, Ara5P-dependent GAA pathway

## Abstract

Ethylene glycol (EG) derived from plastic waste or CO_2_ can serve as a substrate for microbial production of value-added chemicals. Assimilation of EG proceeds though the characteristic intermediate glycolaldehyde (GA). However, natural metabolic pathways for GA assimilation have low carbon efficiency when producing the metabolic precursor acetyl-CoA. In alternative, the reaction sequence catalyzed by EG dehydrogenase, d-arabinose 5-phosphate aldolase, d-arabinose 5-phosphate isomerase, d-ribulose 5-phosphate 3-epimerase (Rpe), d-xylulose 5-phosphate phosphoketolase, and phosphate acetyltransferase may enable the conversion of EG into acetyl-CoA without carbon loss. We investigated the metabolic requirements for *in vivo* function of this pathway in *Escherichia coli* by (over)expressing constituting enzymes in different combinations. Using ^13^C-tracer experiments, we first examined the conversion of EG to acetate *via* the synthetic reaction sequence and showed that, in addition to heterologous phosphoketolase, overexpression of all native enzymes except Rpe was required for the pathway to function. Since acetyl-CoA could not be reliably quantified by our LC/MS-method, the distribution of isotopologues in mevalonate, a stable metabolite that is exclusively derived from this intermediate, was used to probe the contribution of the synthetic pathway to biosynthesis of acetyl-CoA. We detected strong incorporation of ^13^C carbon derived from labeled GA in all intermediates of the synthetic pathway. In presence of unlabeled co-substrate glycerol, 12.4% of the mevalonate (and therefore acetyl-CoA) was derived from GA. The contribution of the synthetic pathway to acetyl-CoA production was further increased to 16.1% by the additional expression of the native phosphate acyltransferase enzyme. Finally, we demonstrated that conversion of EG to mevalonate was feasible albeit at currently extremely small yields.

## 1 Introduction

The use of atmospheric or industry-released CO_2_ as feedstock for production of value-added chemicals is an attractive means to reduce greenhouse gas emissions in a circular economy (Whipple and Kenis, 2010). (Electro)chemical conversion of CO_2_ to methanol or syngas (Goeppert et al., 2014) followed by microbial fermentation is a promising strategy for the biochemical valorization of CO_2_ (Cotton et al., 2020; Gavala et al., 2021; Zhan et al., 2021). However, the technical implementation of microbial production processes using one-carbon (C_1_) substrates remains challenging which is mainly due to the thermodynamically restricted product panel of strictly anaerobic syngas fermentations (Takors et al., 2018; Teixeira et al., 2018), and the extremely high oxygen demand of methanol-based bioprocesses (Frazão and Walther, 2020). The prospection of alternative platform compounds, which can be derived from CO_2_ to serve as substrates in microbial product syntheses, receives therefore increasing attention (Mao et al., 2020; Ma et al., 2022). In this context, the C_2_-compound ethylene glycol (EG), which can be electro-chemically derived from CO_2_ *via* ethylene (Li et al., 2020) and ethylene oxide (Leow et al., 2020) or *via* syngas (Yue et al., 2012) is of particular interest, since it can be converted into a large panel of products without the thermodynamic restrictions observed for anaerobic syngas fermentations, and because EG fermentations have a significantly lower oxygen demand than methanol-based processes. In addition, EG is released upon enzymatic digestion of PET plastic waste (Magalhães et al., 2021). Therefore, biotechnological conversion of EG into high-value added products represents a highly interesting approach, with potential applications in recycling of CO_2_ and up-cycling of plastic waste (Wierckx et al., 2015; Wei and Zimmermann, 2017).

**FIGURE 1:**
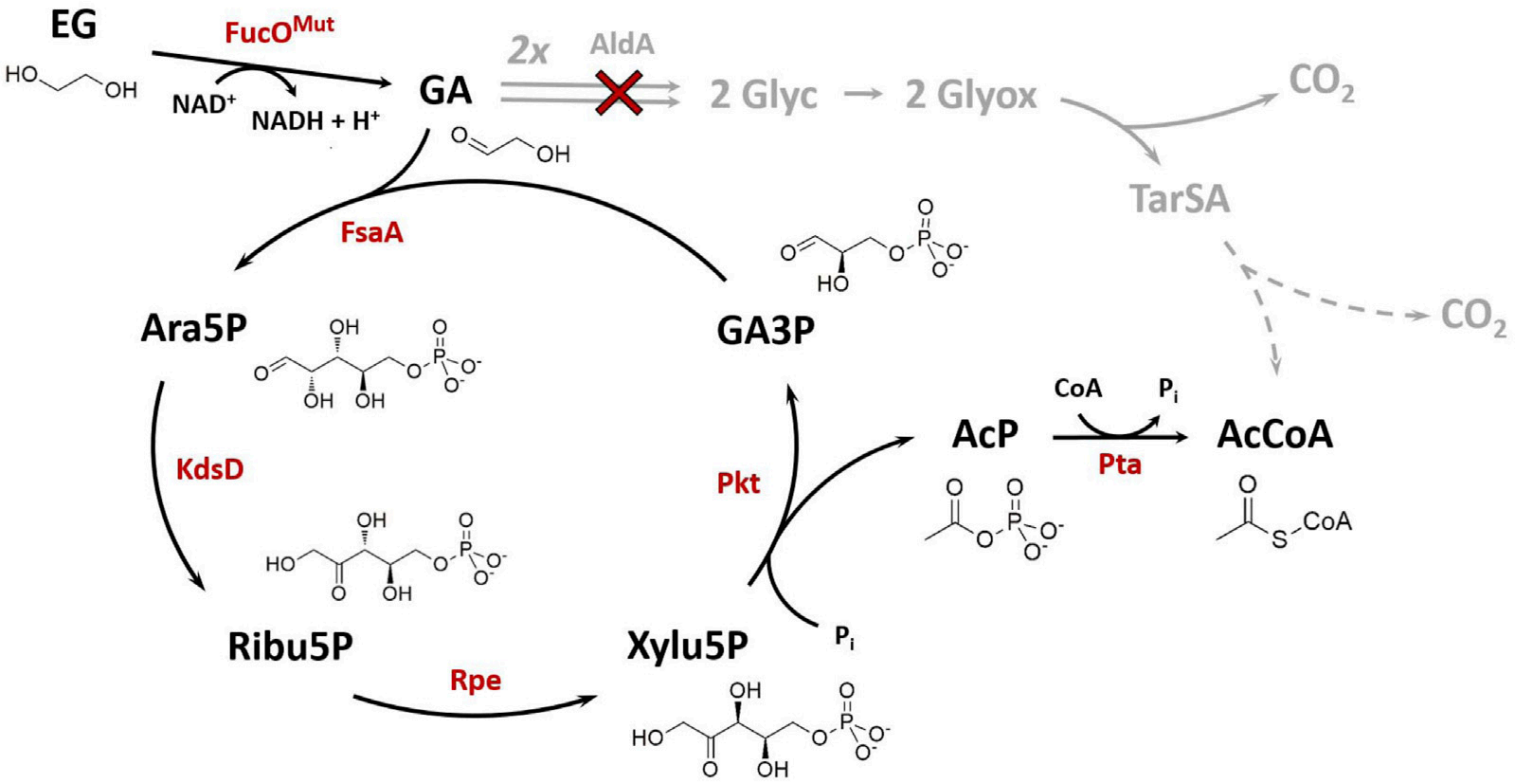
Synthetic Ara5P-dependent GAA pathway (black arrows) for the carbon-conserving conversion of the C_2_ compound ethylene glycol (EG) into acetyl-CoA (AcCoA). Enzymes required for operation of the new route are colored in red. The natural route for glycolaldehyde (GA) assimilation in *E. coli* (grey arrows) was disrupted by deletion of GA dehydrogenase, AldA (red cross). Dashed grey arrows indicate multiple reaction steps between shown intermediates. Arrows indicate the biosynthetic sense of the reactions. Metabolites: AcP, acetyl phosphate; Ara5P, d-arabinose 5-phosphate; GA3P, glyceraldehyde 3-phosphate; Glyc, glycolate; Glyox, glyoxylate; Ribu5P, d-ribulose 5-phosphate; TarSA, tatronate semialdehyde; Xylu5P, d-xylulose 5-phosphate. Enzymes: FucO^Mut^, I6L L7V mutant of native L-1,2-propanediol oxidoreductase; FsaA, arabinose 5-phosphate aldolase; KdsD, d-arabinose 5-phosphate isomerase; Rpe, D-ribulose 5-phosphate 3-epimerase; Pkt, heterologous phosphoketolase; Pta, phosphate acetyltransferase.

Several microbial species are able to use EG as a carbon source for growth (Gonzalez et al., 1972; Kataoka et al., 2001; Mückschel et al., 2012). The natural pathway for EG assimilation proceeds through a sequence of oxidation reactions which yield glyoxylate *via* the intermediates glycolaldehyde (GA) and glycolate (Caskey and Taber, 1981; Mückschel et al., 2012; Franden et al., 2018) (Figure 1). Glyoxylate enters central carbon metabolism either *via* the glyoxylate shunt, or it is converted to 2-phosphoglycerate *via* the intermediates tatronate semialdehyde and d-glycerate (Mückschel et al., 2012; Pandit et al., 2021).

The industrially applied bacteria *Escherichia coli* and *Pseudomonas putida* have been rendered capable of growing on EG using adaptive evolutionary engineering (Boronat et al., 1983; Li et al., 2019). Furthermore, replacing the native tatronate semialdehyde pathway by the heterologous expressed β-hydroxyaspartate cycle (Schada von Borzyskowski et al., 2019) followed by evolutionary engineering also allows growth of *P. putida* on EG and even results in better growth performance (Borzyskowski et al., 2022). In addition to biomass formation, biosynthesis of value-added products such as medium chain length polyhydroxyalkanoates has been achieved using engineered *P. putida* strains (Franden et al., 2018), and our group has developed a synthetic pathway for carbon-conserving conversion of EG to 2,4-dihydroxybutyric acid (Walther and Frazao, 2022). These results underline the potential of using CO_2_ or plastic waste-derived EG as a substrate in microbial production processes. Depending on the target product, however, the carbon efficiency of EG-based microbial product syntheses which employ the natural pathway is not very high. In particular, the conversion of EG into the versatile metabolic precursor acetyl-CoA (AcCoA) suffers from a poor carbon yield which is only 0.5 mol AcCoA per mole of EG (Figure 1). Given that AcCoA serves as a precursor for a wide range of industrially relevant products, including isoprenoids (Martin et al., 2003), 1-butanol (Lian et al., 2014), polyhydroxyalkanoates (Krivoruchko et al., 2015) and fatty acids (Zhang et al., 2011), it becomes clear that more efficient pathways for conversion of EG into AcCoA are highly desireable.

Recently, (Yang et al., 2019) disclosed a new reaction sequence which enables the carbon-conserving conversion of GA into AcCoA. The so called GAA (*glycolaldehyde assimilation*) pathway starts with the initial addition of GA to the acceptor molecule glyceraldehyde 3-phosphate (GA3P) yielding d-arabinose 5-phosphate (Ara5P). The carbon backbone of the phosphorylated sugar is then rearranged to produce d-xylulose 5-phosphate (Xylu5P) by chaining Ara5P isomerase and d-ribulose 5-phosphate epimerase reactions. A phosphoketolase (Pkt) cleaves Xylu5P to regenerate the GA-acceptor GA3P and release acetyl phosphate (AcP) which is eventually converted to AcCoA by a phosphate acetyltransferase. The feasibility of the pathway was demonstrated *in vitro* using isolated enzymatic activities (Yang et al., 2019).

In the present study we extended this work by investigating the *in vivo* functioning of the synthetic pathway. As the number of synthetic pathways proposed for glycolaldehyde assimilation (GAA) continues to grow (e.g., Mao et al., 2021), we refer to the pathway described here as the Ara5P-dependent GAA pathway. An engineered *E. coli* strain was used as host organism to examine the enzymatic requirements for converting EG into the AcCoA-derived product mevalonate (MVA). We used glycerol as the co-substrate for growth and employed ^13^C labeled EG and GA to quantify the contribution of the synthetic pathway to the formation of MVA and characteristic intermediate products. After optimizing expression of the pathway enzymes, we found that 16% of the carbon atoms in the MVA produced in bioconversion experiments using GA as the C_2_ substrate were provided through the synthetic pathway. When GA was replaced by EG, only 0.82% of the final MVA were produced through the synthetic pathway indicating that unfavorable thermodynamics of the NAD-dependent EG dehydrogenase used in this strain severely hampers assimilation of EG.

## 2 Materials and methods

### 2.1 Reagents and chemicals

All chemicals and solvents were purchased from Sigma-Aldrich unless otherwise stated. ^13^C labeled substrates (purity 99% ^13^C-atom) were purchased from Eurisotop, Omicron or Sigma-Aldrich. DNA plasmid isolation and DNA extraction from agarose gels were carried out using kits purchased from New England Biolabs. Genomic DNA of *Clostridium acetobutylicum* (ATCC824) and *Bifidobacterium adolescentis* (ATCC15703) was obtained from the German Collection of Microorganisms and Cell Cultures GmbH (DSMZ, Germany). DNA sanger sequencing was carried out by Microsynth AG (Balgach, Switzerland) or Eurofins SAS (Ebersberg, Germany).

### 2.2 Strains and plasmids

All strains and plasmids used in this study are listed in Table 1 and Table 2.

**TABLE 1:** *Escherichia coli* strains used in this work.

Name	Relevant characteristics	Source
BL21 (DE3)	*huA2 [lon] ompT gal (λ DE3) [dcm]* Δ*hsdSλ DE3 = λ sBamHIo* Δ*EcoRI-B int::(lacI::PlacUV5::T7 gene1) i21* Δ*nin5*	NEB^TM^
MG1655	*F* ^*−*^ *λ* ^*-*^ *ilvG- rfb-50 rph-1*	ATCC
NEB5-α	*fhuA2 Δ*(*argF-lacZ*)*U169 phoA glnV44 Φ80Δ* (*lacZ*)*M15 gyrA96 recA1 relA1 endA1 thi-1 hsdR17*	NEB^TM^
NEB stable	*F′ proA+B+ lacI* ^*q*^ Δ(*lacZ*)*M15 zzf::Tn10* (Tet^R^) Δ(*ara-leu*)*7697araD139 fhuA* ∆*lacX74 galK16 galE15 e14- Φ*80*lacZ*∆*M15 recA1 relA1 endA1 nupG rpsL* (Str^R^) *rph spoT1* ∆(*mrr-hsdRMS-mcrBC*)	NEB^TM^
JW1412	*F* ^*−*^ *λ* ^*-*^ Δ(*araD-araB*)*567* Δ*lacZ4787(::rrnB-3) rph-1* Δ(*rhaD-rhaB*)*568 hsdR51* Δ*aldA776::kan*	CGSC
JW2978	*F* ^*−*^ *λ* ^*-*^ Δ(*araD-araB*)*567* Δ*lacZ4787(::rrnB-3) rph-1* Δ(*rhaD-rhaB*)*568 hsdR51* Δ*yqhD783::kan*	CGSC
C600Zi	*F* ^*−*^ *supE44 hsdR thi-1 thr-1 leuB6 lacY1 tonA21 lacI* ^*q*^ *Spc* ^*R*^	Expressys
EC0	MG1655 Δ*aldA* Δ*yqhD::FRT lacI* ^*q*^ *Spc* ^*R*^	This study
EC1	EC0 *kdsD* ^proD^	This study
EC2	EC0 *rpe* ^proD^	This study
EC3	EC0 *rpe* ^proD^ *kdsD* ^proD^	This study
EG0	EC0 harboring pZA23_MCS	This study
EG1	EC0 harboring pZA23_fucO^Mut^	This study
EG2	EC0 harboring pZA23_fucO^Mut^_Ca-pkt	This study
EG3	EC0 harboring pZA23_fucO^Mut^_fsaA _Ca-pkt	This study
EG4	EC0 harboring pZA23_fucO^Mut^_fsaA_Ca-pkt and pZS13_kdsD	This study
EG5	EC0 harboring pZA23_fucO^Mut^_fsaA _Ca-pkt and pZS13_ rpe	This study
EG6	EC0 harboring pZA23_fucO^Mut^_fsaA _Ca-pkt and pZS13_kdsD_rpe	This study
EG7	EC0 harboring pZA23_fucO^Mut^_Ca-pkt and pZS13_kdsD_rpe	This study
EG8	EC0 harboring pZA23_fucO^Mut^_fsaA_Ca-pkt, pZS33_kdsD_rpe_pta and pMEV-7	This study
GA1	EC0 harboring pZA23_fsaA_Ca-pkt, pZS33_kdsD_rpe and pMEV-7	This study
GA2	EC0 harboring pZA23_talB_Ca-pkt, pZS33_kdsD_rpe and pMEV-7	This study
GA3	EC0 harboring pZA23_talB^F178Y^_Ca-pkt, pZS33_kdsD_rpe and pMEV-7	This study
GA4	EC0 harboring pZA23_fsaA_Ba-pkt, pZS33_kdsD_rpe and pMEV-7	This study
GA5	EC0 harboring pZA23_fsaA_Ca-pkt and pMEV-7	This study
GA6	EC1 harboring pZA23_fsaA_Ca-pkt, pZS33_kdsD_rpe and pMEV-7	This study
GA7	EC2 harboring pZA23_fsaA_Ca-pkt, pZS33_kdsD_rpe and pMEV-7	This study
GA8	EC3 harboring pZA23_fsaA_Ca-pkt and pMEV-7	This study
GA9	EC3 harboring pZA23_fsaA_Ca-pkt, pZS33_kdsD_rpe and pMEV-7	This study
GA10	EC0 harboring pZA23_fsaA_Ca-pkt, pZS33_kdsD_rpe_pta and pMEV-7	This study
GA11	EC3 harboring pZA23_fsaA_Ca-pkt, pZS33_kdsD_rpe_pta and pMEV-7	This study

**TABLE 2:** Plasmids used in this work.

Name	Relevant characteristics	References/origin
pET-28(a)+	*ori f1*, Kan^R^, T7 promoter	Novagen™
pCP20	ori pSC101, Amp^R^, Cm^R^, plasmid expressing Flp recombinase to remove Kan cassette	Cherepanov and Wackernagel (1995)
pMEV-7	*ori colE1*, Amp^R^, P_LlacO_ promoter, *lacI* ^q^, carrying *atoB* from *E. coli*, *mvaS* and *mvaA* from *L. casei*	Xiong et al. (2014)
pKD4	Amp^R^	Datsenko and Wanner (2000)
pKD46	Amp^R^	Datsenko and Wanner (2000)
pZA23	ori *p15A*, Kan^R^, P_A1lacO-1_ promoter	Lutz and Bujard (1997)
pZS13	*ori pSC101*, Amp^R^, P_A1lacO-1_ Promotor	Lutz and Bujard (1997)
pZS33	*ori pSC101*, Cm^R^, P_A1lacO-1_ Promotor	Lutz and Bujard (1997)
pET28_Ca-pkt	pET-28(a)+ carrying *xfp* gene from *C. acetobutylicum*	This study
pET28_fsaA	pET-28(a)+ carrying *fsaA*	This study
pET28_kdsD	pET-28(a)+ carrying *kdsD*	This study
pET28_rpe	pET-28(a)+ carrying *rpe*	This study
pZA23_MCS	pZA23 containing multiple cloning site	Expressys
pZA23_fsaA_Ca-pkt	pZA23 carrying *fsaA* and *xfp* gene from *C. acetobutylicum*	This study
pZA23_talB_Ca-pkt	pZA23 carrying *talB* and *xfp* gene from *C. acetobutylicum*	This study
pZA23_talB^F178Y^_Ca-pkt	pZA23 carrying *talB* ^*F178Y*^ and *xfp* gene from *C. acetobutylicum*	This study
pZA23_fsaA_Ba-pkt	pZA23 carrying *fsaA* and *xfp* gene from *B. adolescentis*	This study
pZA23_fucO^Mut^	pZA23 carrying *fucO* ^*I6L L7V*^	This study
pZA23_fucO^Mut^_Ca-pkt	pZA23 carrying *fucO* ^*I6L L7V*^ and *xfp* gene from *C. acetobutylicum*	This study
pZA23_fucO^Mut^_fsaA_Ca-pkt	pZA23 carrying *fucO* ^*I6L L7V*^, *fsaA* and *xfp* gene from *C. acetobutylicum*	This study
pZS13_kdsD	pZS13 carrying *kdsD*	This study
pZS13_rpe	pZA13 carrying *rpe*	This study
pZS13_kdsD_rpe	pZS13 carrying *kdsD* and *rpe*	This study
pZS33_kdsD_rpe	pZS33 carrying *kdsD* and *rpe*	This study
pZS33_kdsD_rpe_pta	pZS33 carrying *kdsD*, *rpe and pta*	This study

### 2.3 Media

Liquid lysogeny broth (LB) medium (10 g L^−1^ tryptone, 5 g L^−1^ yeast extract and 10 g L^−1^ NaCl) was used for cloning procedures and strain maintenance, protein production and cell recovery from glycerol stocks (25% v/v) kept at –80°C. LB agar plates were prepared by the additional supplementation of 15 g L^−1^ agar-agar. *In vivo* experiments were unless otherwise stated performed in M9 mineral medium (Walther et al., 2017) which contained 18 g L^−1^ Na_2_HPO_4_·12H_2_O, 3 g L^−1^ KH_2_PO_4_, 0.5 g L^−1^ NaCl, 2 g L^−1^ NH_4_Cl, 0.5 g L^−1^ MgSO_4_·7H_2_O, 0.015 g L^−1^ CaCl_2_·2H_2_O, 0.010 g L^−1^ FeCl_3_, 0.012 g L^−1^ thiamine HCl, 0.4 mg L^−1^ NaEDTA·2H_2_O, 1.8 mg L^−1^ CoCl_2_·6H_2_O, 1.8 mg L^−1^ ZnCl_2_SO_4_·7H_2_O, 0.4 mg L^−1^ Na_2_MoO_4_·2H_2_O, 0.1 mg L^−1^ H_3_BO_3_, 1.2 mg L^−1^ MnSO_4_·H_2_O, 1.2 mg L^−1^ CuCl_2_·2H_2_O. This medium was buffered by adding 100 mM 3-(N-morpholino)-propanesulfonic acid (MOPS), adjusted to pH 7 by KOH and filter sterilized. The mineral medium was further supplemented with glycerol as main carbon source (for concentrations, see main text). Whenever required, antibiotics were added at the following concentrations: ampicillin, 100 mg L^−1^; chloramphenicol, 35 mg L^−1^; kanamycin sulphate, 50 mg L^−1^; spectinomycin dihydrochloride pentahydrate, 100 mg L^−1^.

### 2.4 Construction of pET-28(a)+-derived vectors for *in vitro* studies

*In vitro* enzyme studies were conducted using pET-28(a)+ vectors (Novagen) for gene expression*. E. coli* DH5α (New England Biolabs) was used for construction and storage of pET-28(a)+ derived plasmids. Wild-type genes *fsaA, kdsD* and *rpe* were amplified from purified genomic DNA extracted from *E. coli* MG1655, whereas *xfp* gene encoding the Pkt was amplified from genomic DNA of *C. acetobutylicum* ATCC824. The used primers are listed in Supplementary Table S2. After digestion of the resulting DNA fragments by suitable restriction enzymes, T4 DNA ligase (Biolabs) was used to clone target genes into the corresponding sites of pET-28(a)+ vector system. Remaining template DNA was digested by DpnI and the resulting products were transformed into competent cells.

### 2.5 Enzyme expression, purification and quantification

N-His-tagged enzymes were expressed from *E. coli* BL21 (DE3) bearing pET-28a (+) derived plasmids. After an overnight pre-culture in 10 mL LB medium supplemented with kanamycin (37°C, 220 rpm), cells were inoculated at an optical density at 600 nm (OD_600_) of 0.2 in 500 mL shake flasks containing 50 mL LB medium supplemented with kanamycin (37 °C, 220 rpm). To induce protein expression, 1 mM isopropyl-β-D-thiogalactopyranoside (IPTG) was added when an OD_600_ of ∼0.6–0.8 was reached. FsaA*,* Rpe and KdsD enzymes were expressed for 16 h at 20°C, 220 rpm, whilst expression of Pkt from *Clostridium acetobutylicum* (Ca-Pkt) was carried out at 16°C for 30 h, 220 rpm. Afterwards, cell suspensions were centrifuged (at 4,000 g for 10 min, 4°C), the supernatants were discarded and pellets were stored at -20°C until further use.

For protein purification, cell pellets were first thawed on ice and resuspended in 0.75 mL lysis buffer (10 mM HEPES, 300 mM NaCl, pH 7.5). Cells were broken open by three successive rounds of sonication (sonication interval: 10 s, power output: 40%, Topas, UDS 751) and cellular debris was separated from the supernatant at 13,000 g for 10 min, 4°C. The supernatant was then transferred to a new reaction tube to which 75 µL of streptomycin sulfate solution (15 mg mL^−1^) was added. After centrifugation at 13,000 g for 10 min (4°C), the resulting supernatant free of DNA was retained for subsequent steps of protein purification by immobilized metal affinity chromatography (IMAC). A volume of 0.75 mL of TALON^®^ Metal Affinity Resin (Cytiva, USA) was washed twice with 5 volumes of deionized water before being incubated with protein extract on a rotating wheel for 30 min at room temperature. After centrifugation (700 g for 5 min, 4°C), the supernatant was discarded and resin was washed twice with 2 mL of lysis buffer. His-tagged enzymes were then eluted by applying 0.5 mL elution buffer (10 mM HEPES, 300 mM NaCl, 250 mM imidazole, pH 7.5). The eluted fraction was then loaded on an Amicon Ultra-0.5 centrifugal filter unit (pore size, 10 kDa; Merck Millipore, USA) and after a round of centrifugation (14,000 g, 15 min), the buffer was exchanged by applying 0.45 mL lysis buffer and eventually recovering (1,000 g, 2 min) the protein fraction in 0.3 mL of this buffer. Concentration of purified proteins was determined by a Bradford assay (ROTI®Quant, Roth), using bovine serum albumin for preparing a standard calibration curve (0–100 μg mL^−1^).

### 2.6 *In vitro* pathway testing

Prior to demonstrating the *in vitro* functioning of the synthetic pathway, functional enzyme expression was confirmed by measuring activities on corresponding natural substrates as described in Supplementary Information.

Functioning of the whole enzyme cascade was tested in a 1.5 mL reaction tube incubated on a rotary shaker (37°C, 260 rpm), and monitored by offline measurement of formed AcP. The reaction mixture (640 µL) contained 50 mM HEPES (pH 7, adjusted with KOH), 50 mM KH_2_PO_4_ (pH 7, adjusted with KOH), 5 mM MgCl_2_, 1 mM (D)-GA3P, 1 mM thiamine pyrophosphate (TPP). The total protein concentration in the final reaction mix was set to 250 μg mL^−1^. In order to maximize pathway flux, enzyme concentrations were adjusted as followed: FsaA, 60 μg mL^−1^; KdsD, 60 μg mL^−1^; Rpe, 50 μg mL^−1^; Ca-Pkt, 80 μg mL^−1^. Reactions were started by the addition of 10 mM GA. To ascertain pathway function, various controls were performed by replacing either (D)-GA3P, Ca-Pkt, or FsaA by deionized water. To estimate the specific decay kinetics of the unstable AcP, an additional reaction without enzymes and GA but containing 2 mM AcP was included in the analysis. Quantification of AcP was performed *via* its derivatization to iron-acetyl-hydroxamate-complex by the hydroxamate assay (Lipmann and Tuttle 1945). For each time point, 80 µL of sample were transferred to a 96-well plate and the reaction was stopped by the addition of 60 µL of 2 M hydroxylamine solution. After 10 min, 40 µL trichloroacetic acid solution (15% w/v), 40 µL 4 M HCl and 40 µL FeCl_3_·6H_2_O solution (5% w/v in 0.1 M HCl) were sequentially added. The derivatized product was immediately measured spectrophotometrically at 505 nm. For calibration, freshly prepared lithium AcP standard solutions (0–14 mM) were included in reaction mixtures without GA substrate or enzymes.

### 2.7 Construction of pZ-derived vectors for *in vivo* studies

All vectors used and constructed in this study (Table 2) are based on the pZ expression system (Expressys). Backbone vectors used for plasmid construction were pZA23, pZS13 and pZS33, and target genes were cloned into respective multiple cloning site (MCS) module downstream of a P_A1lacO-1_ promoter using NEBuilder^®^ HiFi DNA assembly reaction kit (New England Biolabs). Briefly, genes were amplified by PCR from corresponding genomic DNAs (*fsaA, kdsD, pta, rpe, talB*: *E. coli* MG1655; Ca-*xfp*: *Clostridium acetobutylicum* ATCC824; Ba-*xfp*: *Bifidobacterium adolescentis* ATCC15703) using forward and reverse primer pairs listed in Supplementary Table S2. Primers contained 15- to 20-bp homology arms for subsequent *in vitro* homologous recombination. The forward primer additionally inserted a unique synthetic ribosomal binding site (RBS) in front of gene of interest, which was designed with RBS calculator (Salis et al., 2009). Backbone vectors were linearized by PCR followed by digestion with DpnI. After purification, linearized plasmid and PCR-amplified gene(s) were assembled by using the reaction mix provided in the kit. Resulting plasmids were transformed into NEB^®^ stable cells (New England Biolabs) and verified by DNA sequencing to carry correctly assembled operons. Point mutation in *talB* gene (F178Y) was introduced by PCR using primers listed in Supplementary Table S2.

### 2.8 Strain construction

All strains used and constructed in this study are listed in Table 1 *. E. coli* MG1655 Δ*yqhD* Δ*aldA lacI* ^*q*^ *Spc* ^*R*^ served as reference strain for demonstrating *in vivo* conversion of C_2_ compounds EG and GA. Disruption of *yqhD* and *aldA* genes was achieved by P1vir phage transduction method (Malke, 1993) using single gene deletion mutants from the Keio collection (Baba et al., 2006). The procedure introduces at target locus a cassette (FRT-kan-FRT) composed of kanamycin resistance gene flanked by flippase (Flp) recognition sites. After selection and isolation of antibiotic-resistant clones, gene deletions were confirmed by diagnostic PCR (primer: *yqhD*, TW216, TW217; *aldA*, TW218, TW219;) using DreamTaq polymerase (ThermoFisher Scientific). The kanamycin resistance gene was excised using Flp-recombinase encoded from pCP20 helper plasmid (Cherepanov and Wackernagel, 1995) before a new round of chromosomal modification was initiated. Finally, the *lacI* ^*q*^ locus containing a fused spectinomycin resistance cassette was transduced into producer strains by P1vir phage method using a phage lysate of the donor strain *E. coli* C600Zi strain (Expressys).

For chromosomal overexpression of endogenous *kdsD* and *rpe* genes, native chromosomal 5′-UTR of each gene was replaced by the insulated constitutive promoter proD (Davis et al., 2011) together with a RBS sequence (AGGAGGTATTAT) *via* PCR-mediated λ-Red recombination (Datsenko and Wanner, 2000). The FRT-kan-FRT cassette fused to proD promoter sequence (FRT-kan-FRT-proD) was amplified by PCR (primers: *kdsD*, TW1553 and TW1554; *rpe*, TW 1555 and TW1556; Supplementary Table S2) from the genomic DNA of CF220 strain (kindly provided by Prof. J. M. François from Toulouse Biotechnology Institute, INSA Toulouse, France). The PCR product contained in addition 50 bp flanking sequences which were homologous to the target genomic locus. Recipient *E. coli* MG1655 derived strains expressing λ-Red recombination genes from pKD46 helper plasmid were transformed with ∼400 ng of gel-purified product. After an overnight incubation under non-selective conditions, cell suspensions were plated on LB agar plates supplemented with kanamycin. Successful cassette insertion was confirmed by diagnostic PCR and DNA sequencing of region encompassing promoter and gene of interest. Kanamycin resistance gene was excised as described above. Plasmids were transformed into target *E. coli* strains using standard protocols (Chung et al., 1989).

### 2.9 Shake flask cultivation

All cell cultivation was performed in an orbital shaker (Ecotron, Infors) set at a shaking frequency of 220 rpm. Pre-cultures were started from frozen glycerol stocks and grown at 37°C in 10 mL of LB medium supplemented with appropriate antibiotics until an OD_600_ of ∼2 was reached.

For EG assimilation studies, cells from first pre-culture were diluted by 100-fold in 25 mL of M9 medium containing 220 mM glycerol and appropriate antibiotics. After overnight growth (at 37°C), cells were harvested by centrifugation (4,500 g for 10 min, 4°C), washed once with sterile water and used to inoculate at a starting OD_600_ of 0.2 100 mL shake flasks containing 5 mL of main culture composed of M9 medium supplemented with glycerol (concentrations indicated wherein appropriate in main text) and appropriate antibiotics. EG was added to the medium from the beginning at a final concentration of 500 mM. IPTG (1 mM) was added when cells reached an OD_600_ of 0.6 (∼2.5 h after inoculation), except for MVA production from EG where the optimal induction time was determined to be 2 h after inoculation. Main cultures were carried out at 37°C for acetate production or at 30°C (Xiong et al., 2014) when aiming at MVA biosynthesis. The culture medium used in the experiments to convert EG to MVA contained 100 mM CaCO_3_ as an additional buffer.

In experiments investing GA assimilation, cells from a first pre-culture were used to inoculate main culture (50 mL of 90% v/v M9 medium with 220 mM glycerol and 10% v/v LB in 500 mL shake flasks) with an OD_600_ of 0.2. Cells were cultivated at 30°C and when an OD_600_ of 0.6 was reached, IPTG (1 mM) was added to induce expression of genes under control of P_A1lacO-1_ promoter. Cells were harvested by centrifugation (4,500 g for 10 min, 4°C) at an OD_600_ equal to 1.5, washed twice with sterile water, and resuspended at an OD_600_ of ∼3.5 in 5 mL of M9 medium containing co-substrates as specified in the main text, 1 mM IPTG and 10 mM ^13^C_2_-GA. Samples for extracellular and intracellular metabolites were withdrawn regularly and treated as described in next subsections.

### 2.10 Determination of NAD-dependent EG dehydrogenase activities

EG dehydrogenase activity was measured in strains of *E. coli* MG1655 Δ*yqhD* Δ*aldA lacI* ^*q*^ *Spc* ^*R*^ expressing FucO^Mut^ using a protocol adapted from Boronat et al., 1983. Briefly, cells grown in LB medium were used to inoculate main culture (50 mL of 90% v/v M9 medium with 110 mM glucose and 10% LB in 500 mL shake flasks) with an initial OD of 0.2. Cells were cultivated at 37°C (220 rpm) and 1 mM IPTG was added when an OD_600_ of 0.6 was reached. Biomass was harvested by centrifugation (4,500 g for 10 min, 4°C) at an OD_600_ equal to 1.8, the supernatants were discarded and cell pellets were stored at -20°C until further use. To prepare cell extracts, pellets were thawed on ice, suspended in 25 mM sodium glycine buffer (pH 9.5 by KOH), broken open by three successive rounds of sonication (sonication interval: 10 s, power output: 40%, Topas, UDS 751) and cellular debris was separated from the supernatant at 13,000 g for 10 min, 4°C. Clear supernatants were used in different dilution for further analysis and total protein concentration was determined by a Bradford assay as described above (Section 2.5).

Spectrophotometric assay for EG dehydrogenase activity was performed at 37 °C following NADH formation at 340 nm (ε_340 nm_ = 6.22 mM^−1^cm^−1^). Oxidation activity was measured in an assay mixture that contained 50 mM EG, 100 mM sodium glycine buffer (pH 9.5 by KOH) and 0.5 mM NAD.

### 2.11 Quenching and extraction of intracellular metabolites

At appropriate time intervals, 0.5 mL of cell culture were withdrawn and filtered (polyamide filter, 0.2 µm pore size, Sartorius Stedim) followed by the addition of 1 mL of room temperature deionized water to wash retained cells. Filter and cells were immediately transferred to a 10 mL glass tube containing 5 mL of 75% v/v hot ethanol (80°C). After vortexing for 10 s, glass tubes were incubated for 3 min at 80°C and chilled on ice for an additional 10 min period. The filter was then removed and suspension was centrifuged (13,000 g, 5 min, room temperature) to remove impurities and cell debris. The clear supernatant was stored overnight at -20°C. On the next morning, liquid was removed from the samples at 45°C for 4 h using a benchtop vacuum concentrator (CentriVap Concentrator System, Labconco, USA). Dried metabolites were suspended in 250 µL deionized water and analyzed directly by LC/MS.

### 2.12 Analytical methods

Quantification of extracellular and intracellular metabolites was achieved by high performance liquid chromatography (UltiMate 3,000 system, Dionex, USA), equipped with an autosampler (Dionex, Sunnyvale, USA) keeping samples at 6°C. It was coupled to an UV/vis detector (Dionex, Sunnyvale, USA) measuring at 210 nm and a RI detector (ERC RefractoMax 520, Knauer, Germany). Measurements of ^13^C incorporation was performed by coupling liquid chromatography (Vanquish™, ThermoFisher™, USA) with a mass spectrometer (Q Exactive™ Focus orbitrap, ThermoFisher™, USA).

For analysis of extracellular metabolites, all withdrawn samples were centrifuged (2 min at 13,000 rpm) and syringe-filtered (0.2 µm), and the resulting supernatant was stored at −20°C until analysis. Cell-free supernatant was separated for the analysis of extracellular metabolites using Rezex™ ROA-Organic Acid H^+^ column (300 × 7.8 mm, 8%, Phenomenex) protected by a SecurityGuard™ Carbo H^+^ pre-column (4 × 3 mm, Phenomenex). For detection of glycerol, EG, acetate and MVA analytes were eluted with 2.5 mM H_2_SO_4_ at a constant flow rate of 0.5 mL min^−1^ and a column temperature of 65°C was adjusted. GA, glycolate and d-threose analyses were performed at a column temperature of 35°C using 0.5 mM H_2_SO_4_ as eluent. For LC/MS measurement of ^13^C enrichments in acetate and MVA 0.1% formic acid was used as mobile phase and an isocratic flow of 0.4 mL min^−1^ was adjusted.

For LC/MS-analysis of intracellular metabolites all samples and standards were prepared in 10 mM ammonium acetate (pH 9.2) in a matrix of 60% v/v acetonitrile and 40% v/v water. Separation was achieved by liquid chromatography using a SeQuant^®^ ZIC®-pHILIC column (5 µm polymer 150 × 2.1 mm) with a flow rate of 0.15 mL min^−1^. For an optimal efficiency of separation, a gradient of A (5% ACN, 10 mM ammonium acetate, pH 9.2 adjusted by NH_4_OH) and B (90% acetonitrile, 10 mM ammonium acetate, pH 9.2 by NH_4_OH) was used. The gradient was 0 min, 95% B; 2 min, 95% B; 3 min, 89.4% B; 5 min, 89.4% B; 6 min, 83.8% B; 7 min, 83.8% B; 8 min, 78.2% B; 9 min, 78.2% B; 10 min, 55.9% B; 12 min, 55.9% B; 13 min, 27.9% B; 16 min, 27.9% B; 18 min, 0% B; 23 min, 0% B; 24 min, 95% B; 30 min, 95% B. The temperature of the autosampler was kept at 6°C, injection volume was 5 µL and oven temperature was kept at 25°C. Instrumental settings according to the electrospray ionization were optimized for a flow rate of 0.15 mL min^−1^. Final parameters were adjusted as follows: sheath gas flow rate 32 (arbitrary units), auxiliary gas flow rate 8 (arbitrary units), sweep gas flow rate 0 (arbitrary units), spray voltage -3.5 kV, capillary temperature 250°C and auxiliary gas temperature 200°C. Monoisotopic mass (listed in Supplementary Table S4) and retention time were used to identify metabolites. Retention times of all shown analytes were measured by injecting unlabelled standards. Peak areas were corrected for the contribution of all naturally abundant isotopes using the software IsoCor (Millard et al., 2012).

## 3 Results

### 3.1 The modified synthetic Ara5P-dependent GAA pathway is functional *in vitro*

**FIGURE 2:**
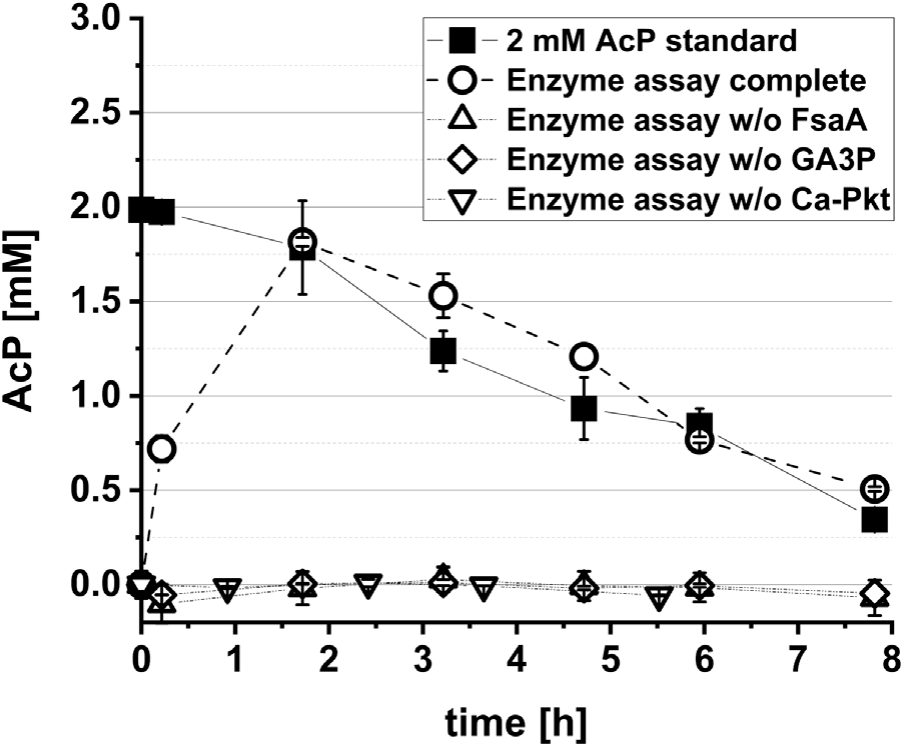
*In vitro* demonstration of the synthetic Ara5P-dependent GAA pathway. The reaction mix contained the his-tag purified enzymes FsaA (60 μg L^−1^), KdsD (60 μg L^−1^), Rpe (50 μg L^−1^) and Ca-Pkt (80 μg L^−1^). Reactions were performed at 37°C in a 1.5 mL reaction tube rotary shaken at 220 rpm. In control reactions either GA3P, Ca-Pkt or FsaA were omitted. Error bars indicate standard error of the mean (*n* = 2).

The aim of this work was the *in vivo* implementation of the synthetic metabolic pathway for the whole-cell bioconversion of EG into AcCoA. Implementation of a synthetic metabolic pathway *in vivo* is much more challenging than its assembly with purified enzymes, since competing side reactions can convert intermediates of the pathway into undesired by-products, and because the activity of individual enzymes cannot be easily changed by simply adding more or less protein. An adequate activity of the enzymes of the target pathway must therefore be achieved, which requires both sufficiently high expression of the corresponding genes in the producer strain and a high specificity of the enzymes with respect to their substrates and products. To meet such endeavour, we introduced changes in selection of pathway enzymes compared to the Yang study (Yang et al., 2019) that first demonstrated the *in vitro* function of the Ara5P-dependent GAA pathway by converting GA into AcP. Specifically, fructose 6-phosphate aldolase (FsaA) from *E. coli* was selected to catalyze the cross aldol addition of GA to GA3P. Unlike the TalB^F178Y^ mutant used by Yang et al., 2019, the FsaA enzyme does not form the byproduct D-ribose 5-phosphate (Schürmann and Sprenger, 2001), which may be deviated into purine *de novo* synthesis. Furthermore, we used the Pkt from *Clostridium acetobutylicum* (Ca-Pkt) instead of the Pkt from *P. stutzeri* (Ps-Pkt). Ca-Pkt has a high preference for Xylu5P over F6P (Bergman et al., 2016) similar to Ps-Pkt. In addition, it was well expressed in *E. coli* ((Servinsky et al., 2012) and own data, not shown). For converting Ara5P into Xylu5P we wanted to recruit the native *E. coli* enzymes Ara5P isomerase (KdsD, Meredith and Woodard 2003) and d-ribulose 5-phosphate 3-epimerase (Rpe, Lyngstadaas et al., 1995) as Yang et al., 2019 (Figure 1). *In vitro* tests were then performed to verify that the synthetic reaction sequence was still functional despite the replacement of two enzymes. Upon incubation of his-tag purified enzymes with 10 mM GA and 1 mM GA3P, a maximum titer of 1.81 ± 0.02 mM AcP was detected, thereby confirming that the pathway is functional and that GA3P can be regenerated (Figure 2).

### 3.2 *In vivo* functioning of the synthetic pathway in *E. coli* requires overexpression of all component enzymes except Rpe

The synthetic reaction sequence is mainly composed of enzymatic activities which are endogenous to *E. coli* and for this reason, we investigated the requirements of overexpressing individual enzymes for *in vivo* pathway operation in this organism. We chose *E. coli* MG1655 *ΔyqhD ΔaldA lacI* ^*q*^ mutant strain (EC0) as host strain, in which genes encoding the major endogenous GA reductase YqhD (Alkim et al., 2015) and GA dehydrogenase AldA (Boronat et al., 1983; Cam et al., 2016) were deleted to support assimilation of GA *via* the synthetic pathway.

**FIGURE 3:**
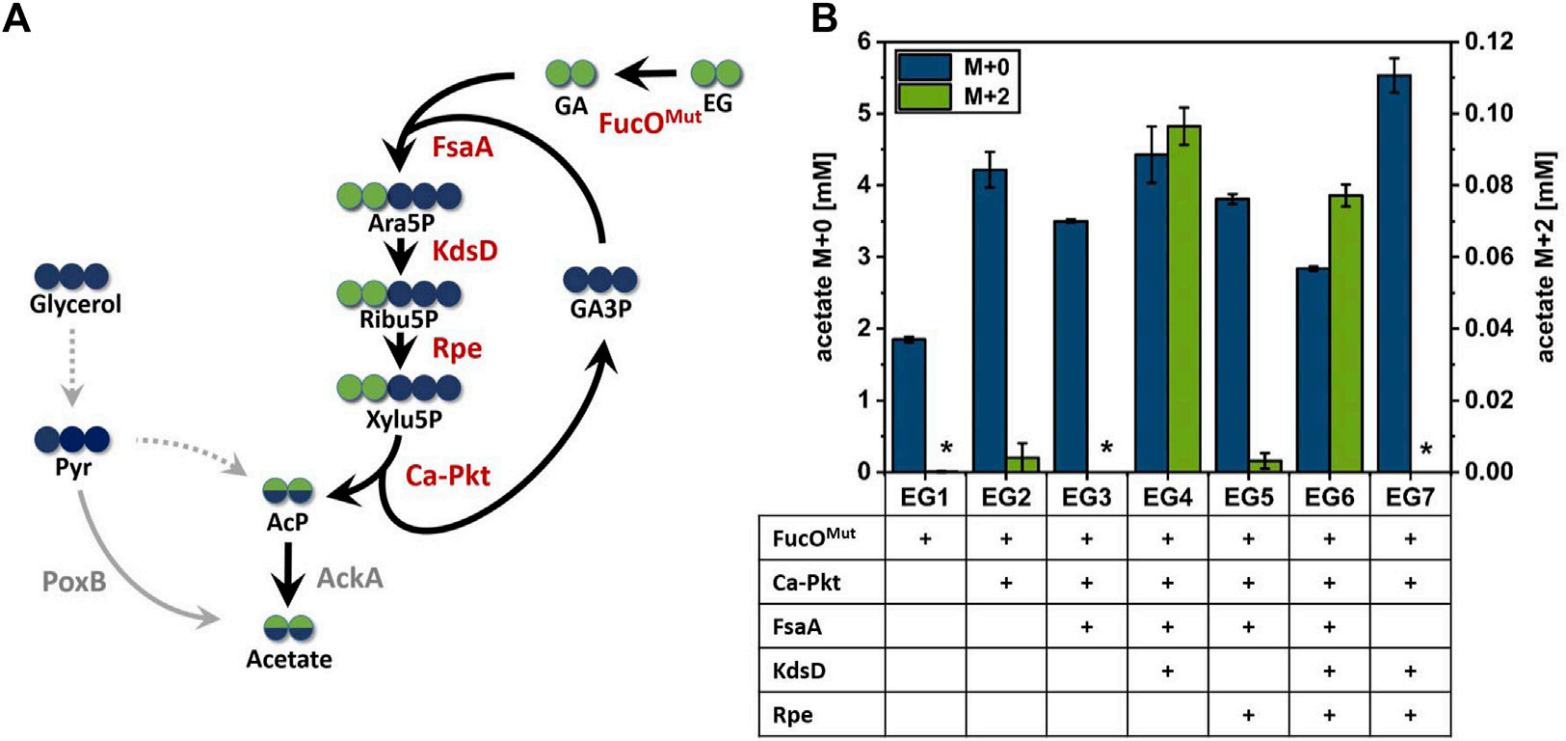
Production of acetate from ^13^C_2_-EG *via* the synthetic Ara5P-dependent GAA pathway. **(A)** Carbon transition from EG into acetate using the synthetic pathway. Green circles represent EG derived carbon, whereas carbon from glycerol co-substrate is shown in blue circles. Arrows indicate the biosynthetic sense of the reactions. **(B)** Maximum detected M+0 and M+2 acetate concentrations during the cultivation of engineered *E. coli* strains overexpressing different enzyme combinations of the synthetic Ara5P-dependent GAA pathway in the presence of unlabeled glycerol (55 mM) and labelled ^13^C_2_ EG (500 mM). Overexpressed enzymes are indicated below the axis (plus sign). Error bars indicate standard error of the mean (*n* = 2). Abbreviations not previously introduced: Pyr, pyruvate; AckA, native acetate kinase; PoxB, native pyruvate oxidase.

Genes encoding the pathway enzymes were expressed from low and medium copy plasmids in different combinations and 55 mM of glycerol (as a growth supporting carbon source) together with 500 mM uniformly labeled ^13^C_2_ EG were fed to the resulting producer strains. Secreted fully labeled (M+2) acetate was used to confirm conversion of ^13^C_2_ EG *via* the Ara5P-dependent GAA pathway (Figure 3A). Glycerol was added as the co-substrate to support growth because *E. coli* produces much less acetate when growing on glycerol than when utilizing glucose (Martínez-Gómez et al., 2012). A comparatively high EG concentration was applied because of the unfavorable thermodynamics of the initial NAD-dependent EG oxidation step (Δ_r_G'° = 23.7 ± 3.9 kJ mol^−1^; a thermodynamic analysis of the entire pathway is shown in Supplementary Figure S1, Noor et al., 2014). As a consequence, it was not possible to reliable quantify the comparatively low EG consumption in these experiments.

It was previously shown that *E. coli* does not naturally express EG dehydrogenase enzymes at sufficient levels even in the presence of this substrate (Pandit et al., 2021). Therefore, the oxygen-insensitive EG dehydrogenase variant FucO^I6L:L7V^ from *E. coli* (Pandit et al., 2021, herein denoted FucO^Mut^) was expressed from pZA23 plasmid in host EC0 strain yielding strain EG1. Adequate expression of the FucO^Mut^ enzyme (0.25 µmol/min/mg_prot_) was verified by an enzymatic assay and acetate production by strain EG1 was monitored. In the presence of EG, strain EG1 showed slightly inhibited growth and significantly increased acetate production compared to reference cultivation without EG (Supplementary Figure S3A). However, LC/MS analysis revealed that the mere expression of EG dehydrogenase did not suffice to secrete fully labeled (M+2) acetate, thus, confirming that this isotopologue cannot be formed by unexpected alternate pathways present in *E. coli*. To understand whether the enhanced secretion of unlabeled acetate was caused by the presence of EG or by the intracellular accumulation of GA, a strain (EG0) carrying an empty plasmid (pZA23_MCS) was cultured under the same conditions. In the presence of EG, strain EG0 accumulated much less acetate than strain EG1 which argues for GA being responsible for increased acetate secretion (shown in Supplementary Figure S2). When the Ca-Pkt enzyme was expressed in addition to FucO^Mut^ (strain EG2), trace amounts of M+2 labeled acetate could be detected which may have been produced through the desired pathway or by direct conversion of GA to AcP catalyzed by a background activity of the Pkt enzyme (Yang et al., 2019). We continued our analysis by constructing strain EG3 which overexpressed the Ara5P aldolase FsaA in addition to the EG dehydrogenase and Xylu5P Pkt enzymes. Much to our surprise, this strain did not produce M+2 acetate at detectable levels which indicated that the pathway was not operational despite the fact that KdsD and Rpe are constitutively expressed in *E. coli* (Lyngstadaas et al., 1998; Meredith and Woodard 2005).

**FIGURE 4:**
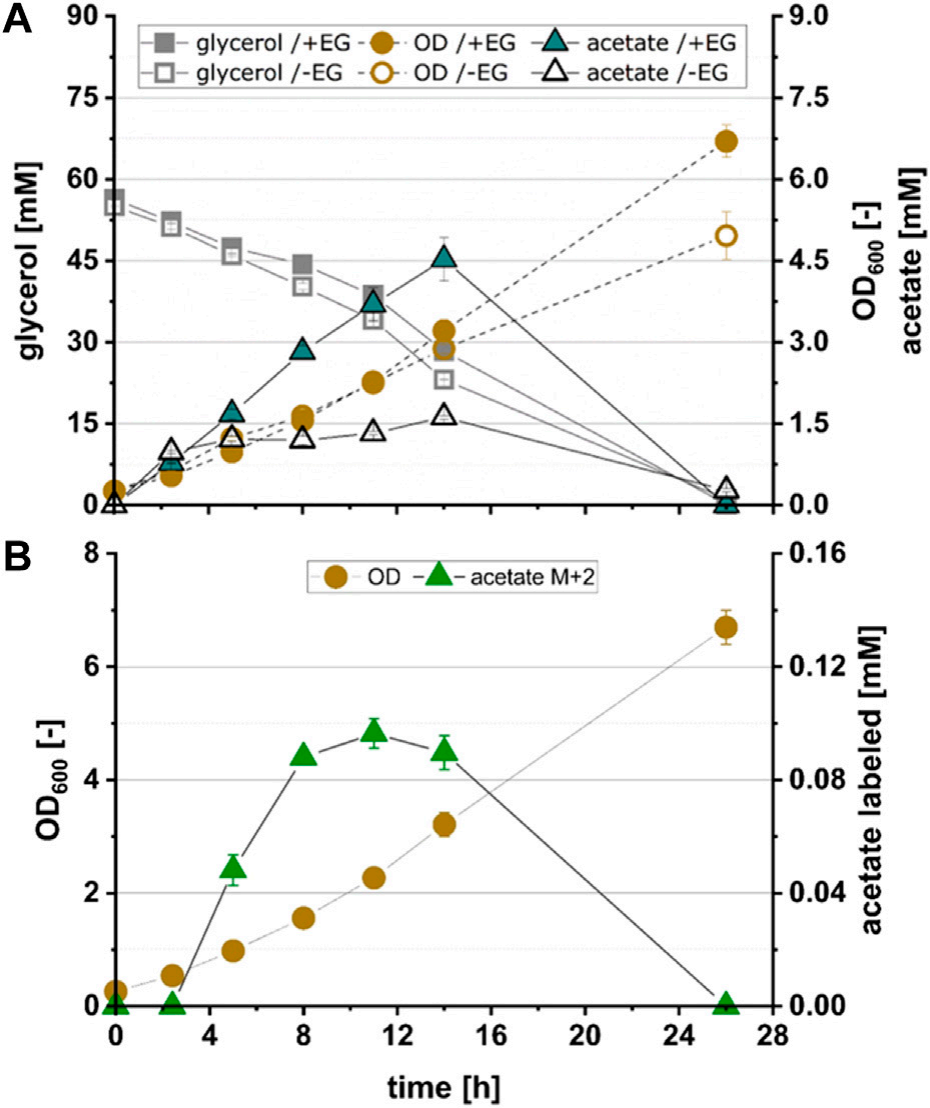
Growth and product kinetics of strain EG4 (*E. coli* Δ*yqhD* Δ*aldA lacI* ^*q*^ + pZA23_fucO^Mut^_fsaA_Ca-pkt + pZS13_kdsD) for the conversion of acetate from ^13^C_2_-EG. **(A)** Time course of optical density (OD_600_), glycerol concentration and total acetate concentration during cultivation of EG4 expressing all enzymes of the Ara5P-dependent GAA pathway with exception of Rpe in the presence (+EG) and absence (-EG) of EG. **(B)** Time course of labelled acetate fraction M+2 during cultivation of EG4. All data refer to experiments performed in minimal medium (M9) supplemented with 55 mM glycerol and 500 mM ^13^C_2_ EG. Error bars indicate standard error of the mean (*n* = 2).

To test whether pathway function was hampered by insufficient expression of KdsD and/or Rpe, strains EG4, EG5 and EG6 were constructed in which either KdsD (EG4), Rpe (EG5) or both enzymes (EG6) were overexpressed in addition to FucO^Mut^, FsaA and Ca-pkt. Strain EG4 showed production of M+2 acetate in significant quantities (Figure 3B). The fraction of fully labeled acetate increased directly after induction of protein synthesis (2.4 h) and reached a maximum after 11 h (see Figure 4). Even though the M+2 fraction accounted only for 2.6% of the total amount of acetate formed by strain EG4, this result clearly demonstrated *in vivo* functioning of the synthetic pathway. In contrast to strain EG4, only trace amounts of M+2 acetate were detected during the cultivation of EG5 confirming that native expression of KdsD is not sufficient to ensure flux *via* the synthetic pathway. Simultaneous overexpression of KdsD and Rpe in strain EG6 overexpressing all enzymes from EG to AcP did not produce more M+2 acetate than strain EG4, indicating that activity of chromosomally expressed Rpe did not limit pathway flux under these conditions. Finally, we investigated the role of FsaA, and constructed strain EG7, which harbored plasmids encoding genes for all enzymes with exception of FsaA. Again no formation of M+2 acetate was observed for this strain. In summary, these results indicate that all component enzymes of the Ara5P-dependent GAA pathway except for Rpe need to be overexpressed in *E. coli* to convert the C_2_ alcohol EG into the intermediate AcP and further into acetate.

### 3.3 The AcCoA-derived product mevalonate can be synthesized *in vivo* from GA

While fixation of EG *via* the synthetic metabolic pathway was confirmed, acetate formation on EG was found to be extremely low (max. 0.096 mM acetate in presence of 500 mM EG; strain EG4). We attributed this observation to the thermodynamic hurdle in the NAD-dependent EG oxidation to GA (compare Section 3.2), which hampers carbon flux into the pathway. Therefore, we used the product of EG oxidation, i.e., GA, as the substrate for further investigations. Previously performed work confirms that uptake of this aldehyde is possible without additional expression of an importer (Frazão et al., 2018; Lu et al., 2019). AcCoA could not be reliably measured by our LC/MS method. To demonstrate that the pathway works up to the level of this central precursor, we installed a heterologous pathway to produce the stable isoprenoid-precursor MVA, which is exclusively derived from AcCoA (Xiong et al., 2014). The fraction of labeled carbon in extracellular MVA could therefore be used as a proxy for estimating the contribution of the synthetic pathway to the total biosynthesis of AcCoA. Xiong et al., 2014 previously demonstrated that production of MVA was possible in *E. coli* when AcCoA acetyltransferase from *E. coli* (AtoB), and heterologous acetoacetyl-coenzyme A thiolase and MVA synthase activities from *Lactobacillus casei* (Lc-MvaS and Lc-MvaE, respectively) were (over)expressed. Accordingly, a plasmid harboring the MVA pathway genes (pMEV-7, Xiong et al., 2014) was transformed into the strain which operated the Ara5P-dependent GAA pathway starting from GA.

Strain GA1, which was derived from the *E. coli* host strain EC0 (*E. coli* MG1655 *ΔyqhD ΔaldA lacI* ^*q*^) and expressed the MVA pathway as well as all genes constituting the Ara5P-dependent GAA pathway except for EG dehydrogenase, was selected as the starting point of our investigations. Anticipating an increase in flux when using GA instead of EG as the substrate and in an attempt to prevent any potential limitation of carbon flux through the pathway, we decided to use a strain which also overexpressed Rpe even though its overexpression did not yield any improvements when producing acetate from EG.

*E. coli* did not grow at elevated GA concentrations. Since an initial concentration of 10 mM GA was previously shown to be suitable for studying the *in vivo* function of a GA-dependent pathway in *E. coli* (Lu et al., 2019; Walther and Frazao, 2022), we chose this concentration to investigate MVA production. In these bioconversion experiments, cells were induced by 1 mM IPTG in the pre-culture, harvested and resuspended in fresh M9 medium containing 1 mM IPTG and 10 mM of fully labeled ^13^C_2_-GA adjusting to an OD_600_ of 3.5. The fraction of GA-derived carbon in extracellular metabolites was quantified using LC/MS analysis. When GA was the only carbon source, no MVA was detected in the supernatant after 24 h of incubation. However, detection of 0.21 mM fully labeled (M+2) acetate indicated that conversion of GA to the intermediate AcP *via* the synthetic pathway was functional (Table 3). The conversion of AcCoA to MVA requires two NADPH molecules which cannot be provided if GA is used as the substrate and no carbon is channeled away from the pathway into NADPH-yielding reactions. Therefore, we investigated whether the addition of a co-substrate could increase AcCoA and MVA production through the synthetic pathway and supplemented the cultures with unlabeled glucose (5 mM) or glycerol (10 mM) at an equal C-mole basis. Both in the presence of either glucose and glycerol, again fully labeled acetate accumulated in the first 2 h of incubation, but was consumed after co-substrate depletion (Supplementary Figure S4). The maximum concentrations of M+2 acetate for glucose and glycerol were 0.12 ± 0.08 mM and 0.46 ± 0.17 mM, respectively. Addition of either carbon source enabled the formation of MVA. When glycerol was used as the co-substrate, the fraction of GA-derived carbon in MVA amounted to 12.42%, which was significantly higher than during utilization of glucose (5.44%). Although this result showed that the assimilation of GA made a significant contribution to the biosynthesis of AcCoA and MVA, it is important to note that MVA was only produced in small quantities (0.28 mM), which accounted only for ∼ 1% [C-mol] of the consumed GA and ∼ 5% [C-mol] of the consumed glycerol. Major fermentative by-products were EG and d-threose (Table 3). Thus, reduction of GA to EG by unspecific GA reductases (Alkim et al., 2015), and the Fsa-catalyzed threose-yielding homo-aldol addition of GA (Garrabou et al., 2009) were the major reactions which drained GA-derived carbon into metabolic by-products. To further investigate why the balance of GA-derived carbon did not close (recovery was only ∼50%, Table 3), we incubated 10 mM of GA in glycerol-containing M9 medium without cells and measured the GA concentration after 24 h. We found that approximately 30% of the initially added GA was consumed in spontaneous abiotic reactions yielding unidentified products. However, even if loss through abiotic reactions would be considered in the balance, a significant portion of GA-derived carbon (∼20%) still could not be recovered.

**TABLE 3:** Biosynthesis of mevalonate (MVA) and metabolic by-products during incubation of strain GA1 (*E. coli* Δ*yqhD* Δ*aldA lacI* ^*q*^ + pZA23_fsaA_Ca-pkt + pZS33_kdsD_rpe + pMEV-7) on ^13^C_2_-GA. The medium contained 1 mM IPTG, 10 mM ^13^C_2_-GA, and was supplemented with 5 mM glucose or 10 mM glycerol as indicated. Formation of by-products and recovery of GA-derived carbon was estimated after 24 h of incubation at 30°C. Cell-free M9 medium containing GA and glycerol served as control. Corresponding fermentation kinetics are shown in Supplementary Figure S4. Experiments were performed duplicate (*n* = 2).

Condition	Remaining GA [mM]	Total MVA [mM]	Relative ^13^C enrichment in MVA [%]	M+2 acetate [mM]	Side products derived from GA [mM]		GA derived carbon recovery [%]
					EG	Threose	
10 mM GA	4.80 ± 0.08	—	—	0.21 ± 0.00	0.97 ± 0.01	0.79 ± 0.01	74.81 ± 0.31
10 mM GA + 5 mM glucose	—	0.32 ± 0.01	5.44 ± 0.02	—	3.54 ± 0.06	0.51 ± 0.02	47.09 ± 0.19
10 mM GA + 10 mM glycerol	—	0.28 ± 0.03	12.42 ± 0.85	—	4.43 ± 0.31	0.61 ± 0.01	48.48 ± 2.75
Cell free10 mM GA + 10 mM glycerol	6.96 ± 0.05	—	—	—	—	—	69.93 ± 2.16

### 3.4 Carbon tracing experiments verify functioning of synthetic pathway and identify competing metabolic pathways

**FIGURE 5:**
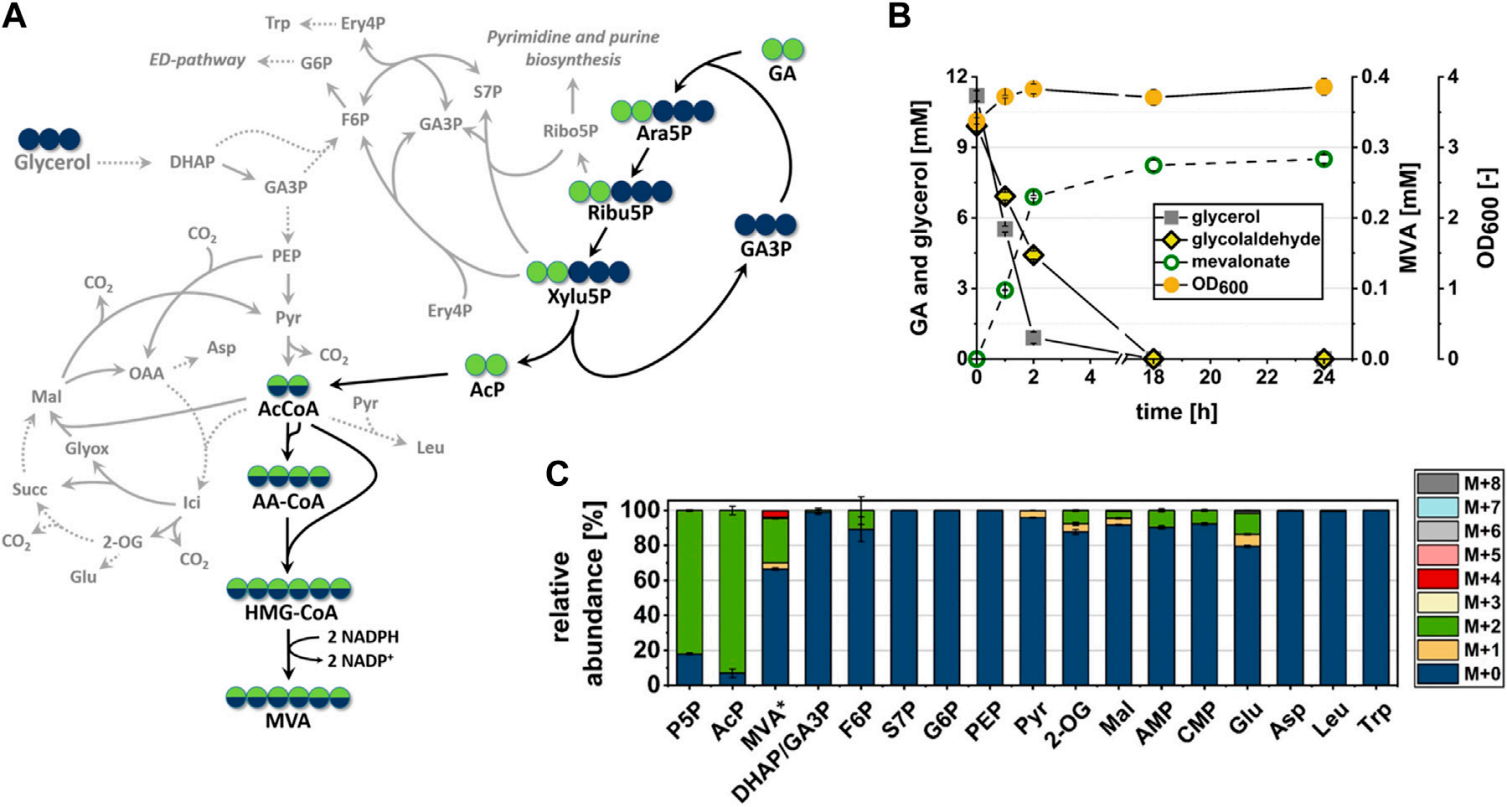
*In vivo* conversion of the C_2_-compound GA into MVA by strain GA1 (*E. coli ΔyqhD ΔaldA lacI* ^*q*^ + pZA23_fsaA_Ca-pkt + pZS33_kdsD_rpe + pMEV-7). Cells were incubated in minimal M9 medium supplemented with 1 mM IPTG, 10 mM glycerol and 10 mM ^13^C_2_-GA at 30°C. Error bars indicate standard error of the mean (*n* = 2). **(A)** Carbon transition during GA assimilation *via* synthetic pathway in cellular context. Dashed lines imply more than one involved reaction between shown intermediates. Blue or green filled circles indicate that the carbon atom may be derived from glycerol or GA, respectively. Black arrows indicate the proposed reaction sequence from GA into MVA in the biosynthetic direction. Grey lines refer to side reactions by enzymes involved in the central carbon metabolism. **(B)** Substrate consumption, MVA production and biomass formation during incubation time. **(C)** Relative abundance of isotopologues of Ara5P-dependent GAA pathway intermediates and metabolites of the central carbon metabolism after 1 h of incubation. Relative abundance was measured from extracts of intracellular metabolites with the exception of MVA, which was measured in the supernatant (extracellular). P5P sugars (which are Ara5P, Ribu5P, Xylu5P and D-ribose 5-phosphate) and GA3P/DHAP are shown as pools since they could not be separated by LC/MS. Abbreviations not previously introduced: 2-OG, 2-oxoglutarate; AA-CoA, acetoacetyl-CoA; AMP, adenosine monophosphate; Asp, aspartate; CMP, cytosine monophosphate; DHAP, dihydroxyacetonphosphate; ED, Entner-Doudoroff; Ery4P, d-erythrose 4-phosphate; F6P, d-fructose 6-phosphate; G6P, D-glucose 6-phosphate; Glu, glutamate; HMG-CoA, 3-hydroxy-3-methylglutaryl-CoA; Ici, iso-citrate; Leu, leucine; Mal, malate; MVA*, extracellular mevalonate; PEP, phosphoenolpyruvate, Ribo5P, ribose 5-phosphate, Trp, tryptophan.

The fact that the fate of at least 20% of the GA-carbon could not be explained by spontaneous degradation or conversion to extracellular products indicated the presence of additional metabolic side reactions which have diverted carbon away from the target pathway. To verify that the presence of labeled carbon in AcCoA and MVA could indeed be explained by the operation of the synthetic pathway, and to identify competing metabolic routes (see Figure 5A) which may have contributed to consumption of GA-derived carbon, we analyzed the distribution of isotopologues in metabolites of central metabolism. Samples of intracellular metabolites were taken at 1 h after incubation with fully labeled ^13^C_2_-GA and unlabeled glycerol (data shown in Figure 5C). At this time point, when cells were not limited for neither glycerol nor GA (see Figure 5B), we found more than 80% of the measured P5P pool (which includes Ara5P, Ribu5P, Xylu5P and D-ribose 5-phosphate/Ribo5P) to be labeled in two carbon atoms (M+2). In addition, most of the intracellular AcP (∼90%) was fully labeled (M+2), whereas the GA-accepting molecule GA3P and the glycolytic metabolites phospho*enol*pyruvate and pyruvate contained very little to no GA-derived carbon (Figure 5C). Together, these findings indicated that the intended synthetic reaction sequence was functional *in vivo* and that it was the only source of GA-derived carbon in AcCoA and MVA.

The Ara5P-dependent GAA pathway shares the intermediates Xylu5P and Ribu5P with the natural central carbon metabolism of *E. coli* (Figure 5A). To evaluate whether the apparent loss of GA-derived carbon (compare Section 3.3) could be explained by the consumption of these compounds through competing native pathways, the isotopologue distribution of adjacent metabolites was investigated. Indeed, we could detect a ∼10% (M + 2) fraction in the fructose 6-phosphate (F6P) pool, which argued for a transketolase-dependent carbon transition between Xylu5P and F6P. However, since there was no labeled carbon in neither glucose 6-phosphate nor GA3P, we concluded that this route did not contribute significantly to the overall carbon balance. Similarly, the absence of ^13^C-label in sedoheptulose 7-phosphate and tryptophan indicated that transaldolase did not divert carbon away from the synthetic pathway (Figures 5A, C). The presence of a ∼10% M + 2 fraction in both adenosine monophosphate and cytosine monophosphate showed that purine and pyrimidine *de novo* synthesis drained Ribu5P from the target pathway. But since the biomass concentration did not increase much during the experiment, it is likely that carbon consumption through these metabolic routes was also negligible. While these results suggested that carbon loss in the pathway section between GA and AcCoA was small, presence of (M+2) isotopologue fractions in 2-oxoglutarate (7.6%) and malate (4.1%) showed that a significant portion of AcCoA and therefore GA-derived carbon was channeled through the TCA cycle, thus being lost in the form of CO_2_.

Taken together, we observed efficient incorporation of GA-derived carbon along the proposed pathway with high (over 80%) proportions of M+2 isotopologues in all intermediates. From the near absence of labeled carbon in pyruvate and the labeling pattern of MVA (Figure 5C) it can be concluded that 13.55% of the intracellular AcCoA were produced through the synthetic pathway during the first hour of incubation. However, a large fraction of the AcCoA was not converted to the target product MVA but most likely entered the TCA cycle as witnessed by the low overall MVA yield and the presence of significant fractions of GA-derived carbon in TCA cycle metabolites.

### 3.5 Flux *via* the Ara5P-dependent GAA pathway can be increased by overexpression of phosphate acetyltransferase

**FIGURE 6:**
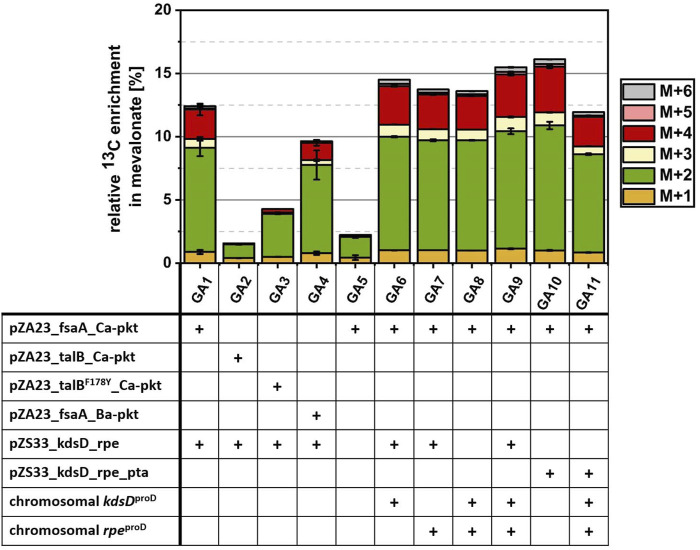
Investigation of various metabolic optimization approaches for the conversion of ^13^C_2_-GA into MVA. Cells were incubated in M9 medium supplemented with 1 mM IPTG, 10 mM glycerol and 10 mM ^13^C_2_ GA at 30°C. Strain GA1 (*E. coli ΔyqhD ΔaldA lacI* ^*q*^ + pZA23_fsaA_Ca-pkt + pZS33_kdsD_rpe + pMEV-7) was used as reference strain. Using strain EC0 (*E. coli* ΔyqhD ΔaldA lacI^q^) expressing the MVA pathway from the pMEV-7 plasmid as the starting point, different enzymes of the synthetic Ara5P-dependent GAA pathway were overexpressed either from plasmids or by replacing the native promoter of the native genes in the chromosome by the strong constitutive proD promoter. Genetic modifications (shown in table) of each strain are marked with a plus sign. Relative enrichment of ^13^C-carbon found in the product MVA after 24 h of incubation was calculated by dividing amount of labelled C-atoms by total amount of C-atoms summed up for each detected isotopologue fraction of MVA. Error bars indicate standard error of the mean (n ≥ 2).

With the aim of achieving a higher metabolic flux *via* the Ara5P-dependent GAA metabolic pathway, various optimization approaches were pursued starting from reference strain GA1 (*E. coli* Δ*yqhD* Δ*aldA lacI* ^*q*^ *+* pZA23_fsaA_Ca-pkt + pZS33_kdsD_rpe + pMEV-7*,* Table 3 and Figure 5). The impact of different genetic modifications was quantified by comparing the relative amount of labeled carbon found in the extracellular product MVA after 24 h of incubation with 10 mM ^13^C_2_ GA and 10 mM glycerol (Figure 6).

Strain GA1 expressed the Ara5P aldolase FsaA, which was chosen on the basis of its high specificity for the desired product. However, Yang et al., 2019 used an alternative aldolase TalB^F178Y^ (Rale et al., 2011) to demonstrate the *in vitro* functioning of the Ara5P-dependent GAA pathway. To check whether the use of TalB^F178Y^ could improve the *in vivo* conversion of GA, we replaced the FsaA encoding gene on the medium-copy number plasmid by either the wildtype TalB or the mutant TalB^F178Y^ gene resulting in strain GA2 and GA3, respectively. While GA1 showed an incorporation of 12.42% GA-derived carbon after 24 h, it was found that in strains GA2 and GA3 only 1.56% and 4.28% of the MVA was derived from GA (Figure 6). This observation confirmed FsaA Ara5P aldolase as a superior enzyme in the *in vivo* context.

In the next step, we investigated whether the replacement of the Ca-Pkt by the Pkt from *Bifidobacterium adolescentis* (Ba-Pkt) could improve pathway function. The Ba-Pkt is a well-characterized enzyme and has already been used in *E. coli* to construct a synthetic non-oxidative glycolysis (Bogorad et al., 2013; Lin et al., 2018). Strain GA4 carried a pZA23 plasmid which harbored Ba-*pkt* instead of the Ca-*pkt* gene. The MVA produced by GA4 contained less GA-derived carbon (9.46%) than the GA1 reference strain, demonstrating that Ca-Pkt is the better pathway enzyme, presumably, because Ba-Pkt has a lower specificity for Xylu5P (Bergman et al., 2016).

We next set out to investigate the impact of overexpressing KdsD and/or Rpe on flux through the synthetic pathway. To this end, the corresponding genes were overexpressed from a plasmid and/or the chromosome by replacing the natural promoter of the two genes by the synthetic constitutive strong promoter ProD (Davis et al., 2011). As expected from the results of the EG-to-acetate conversion experiments, only a very small fraction of MVA (2.2%) could be derived from GA when neither of the two enzymes was overexpressed in strain GA5. In contrast, the simultaneous plasmid- and chromosomally-based overexpression of both genes (strain GA9) resulted in a^13^C-enrichment of MVA (15.49%) which was increased by 24.7% compared to that observed for strain GA1 (12.42%, which overexpresses Rpe and KdsD from plasmid alone).

Based on the analysis of intracellular metabolites (strain GA1, Figure 5C), a strong discrepancy between labeling in the communicating metabolite pools AcCoA/MVA and AcP was detected. Possible explanations for a much lower ^13^C content in AcCoA/MVA are a low flux *via* the Ara5P-dependent GAA metabolic pathway or an insufficient phosphate acetyltransferase (Pta) activity. To test whether this enzyme activity was a bottleneck, we extended the genetic repertoire of strain GA1 by overexpressing Pta from the low-copy number pZS33 plasmid in addition to KdsD and Rpe. The resulting strain GA10 produced MVA containing a fraction of 16.12% GA-derived carbon which corresponded to an increase of 28.8% compared to the reference strain GA1. This result showed that endogenous expression of Pta enzyme had a limiting effect on the flux between AcP and AcCoA, which seems surprising given that this enzyme is highly active and reported to be constitutively expressed (Campos-Bermudez et al., 2010; Martínez-Gómez et al., 2012; Enjalbert et al., 2017). Finally, we tried to combine the positive effect observed when overexpressing Pta, KdsD and Rpe from the pZS33 plasmid with boosting the expression of KdsD and Rpe from the chromosome. However, the corresponding strain GA11 exhibited a strong growth phenotype (not shown) and a severe drop of the GA-derived carbon fraction in MVA (11.94%, Figure 6). Taken together, these results indicate that the synthetic pathway can make a significant contribution to the biosynthesis of AcCoA. To further enhance its performance, the expression of the constituent genes needs to be carefully tuned and a better understanding of the apparent limiting role of Pta is required.

### 3.6 The AcCoA-derived product mevalonate can be synthesized from EG *via* the synthetic pathway

The metabolic pathway was designed to enable the carbon-conserving conversion of EG to AcCoA-derived products. The assimilation of EG through the pathway has already been demonstrated by detection of EG-derived acetate. Since the plasmid-borne overexpression of the native enzyme Pta proved advantageous for MVA production from GA, the metabolic setting of strain GA10 (which is *E. coli* Δ*yqhD* Δ*aldA lacI* ^*q*^ *+* pZA23_fsaA_Ca-pkt + pZS33_kdsD_rpe_pta + pMEV-7) was extended for the bioconversion of EG into MVA by the additional expression of the EG dehydrogenase FucO^Mut^ (strain EG8 = *E. coli* Δ*yqhD* Δ*aldA lacI* ^*q*^ *+* pZA23_fucO^Mut^_fsaA_Ca-pkt + pZS33_kdsD_rpe_pta + pMEV-7). To investigate the ability of the engineered strain to convert EG into MVA, it was cultured in the presence of 500 mM ^13^C_2_-EG. An increased concentration (250 mM) of the growth-supporting co-substrate glycerol was added to extend growth and production phase. As mentioned above, by using a high EG concentration it was possible to overcome the thermodynamically unfavorable initial EG oxidation step, but EG uptake remained too small for a reliable quantification. Corresponding growth and production kinetics are shown in Supplementary Figure S5A. No accumulation of GA nor threose was detected during the whole cultivation. Sampling after complete glycerol consumption (46 h) showed a total MVA concentration of 10.80 ± 0.65 mM. LC/MS analysis revealed a relative ^13^C enrichment in MVA of 0.82% in the final sample and a maximum of only 1.24% (Supplementary Figure S5B) after 22 h of cultivation. However, these results indicating that the synthetic pathway was functional albeit at currently extremely small yields.

## 4 Discussion

EG is an attractive carbon source for microbial product syntheses since it can be derived from plastic waste or CO_2_. In our work, we investigated the metabolic requirements for implementing in *E. coli* the previously suggested carbon-efficient Ara5P-dependent GAA pathway (Yang et al., 2019) for converting EG into AcCoA. The reaction sequence suggested by (Yang et al., 2019) was modified by replacing the Ara5P aldolase (FsaA instead of TalB^F178Y^) and using a different Pkt (Ca-pkt instead of Ps-Pkt). *In vitro* synthesis of AcP from GA and turnover of GA3P confirmed the functionality of the modified enzyme cascade. However, the enzyme cascade had a low yield converting only 18.1% of the provided GA into AcP. The most likely explanation for the poor performance is a rapid aldehyde-induced inactivation of one or more enzymes *in vitro*, which was also suggested in the study of (Yang et al., 2019). In addition, the unstable GA3P metabolite (Sánchez-Moreno et al., 2012) may have been degraded over time. Since the focus in this work was not on the optimization of the *in vitro* enzyme cascade but on the *in vivo* implementation of the pathway, we did not pursue additional optimization attempts regarding the enzyme cascade.

By feeding ^13^C-labelled EG to cells and monitoring the labelling pattern of the secreted acetate we further showed that the pathway is functional *in vivo* up to the acetate precursor AcP, provided that at all component enzymes but Rpe are overexpressed. It is of note that all strains expressing components of the synthetic pathway exhibited an up to three-fold higher acetate secretion in the presence of EG than in the absence of the C_2_ compound (Supplementary Figure S3). Only by using carbon tracing experiments it was possibly to reveal that this increase of acetate was not due to an EG-derived carbon flux through the synthetic pathway, which contributed only approximately 2.6% to the overall acetate production. Instead, our results suggest that accumulation of intracellular GA was a major cause for unscheduled acetate formation (compare fermentation kinetics of EG0 and EG1, Supplementary Figure S2 and Supplementary Figure S3A).

We next set out to investigate the functioning of the pathway up to AcCoA by investigating the biosynthesis of MVA, which is exclusively derived from AcCoA. To bypass the thermodynamic obstacle of the initial EG oxidation, we investigated the conversion of GA to MVA using glycerol again as the co-substrate. While our strains indeed synthesized MVA from both substrates, this production was very low and cannot be compared to other studies which reported highly efficient biosynthesis of this compound (Wang et al., 2016). It should be noted, however, that our work is the first which actually demonstrates production of a value-added compound *via* a synthetic AcCoA-producing GAA pathway (Lu et al., 2019; Yang et al., 2019; Mao et al., 2021). In addition, the focus of our work was not on optimizing MVA production but on using the labeling pattern of extracellular MVA to reliable quantify the contribution of the synthetic pathway to the biosynthesis of AcCoA (which could not be measured by our LC/MS method). We showed that replacing the Ara5P aldolase TalB^F178Y^, which was previously applied in the *in vitro* enzyme cascade (Yang et al., 2019), by FsaA was crucial for improving *in vivo* performance of the pathway. This result can most likely be explained by the superior specificity of FsaA which only produces Ara5P from GA3P and GA (Schürmann and Sprenger, 2001) while the TalB^F178Y^ mutant also produces Ribo5P (Yang et al., 2019). Although Ribo5P can be converted to Ribu5P by ribose 5-phosphate isomerase (Yang et al., 2019), the isomerase reaction competes for its substrate with transketolase (Sprenger et al., 1995) and PRPP synthesizing (D)-ribose-phosphate pyrophosphokinase (Hove-Jensen, 1985), which may deviate carbon away from the target pathway. In contrast, the FsaA catalyzed (Garrabou et al., 2009) formation of threose from GA (see Table 3) is reversible and, to the best of our knowledge, no threose-degrading enzymatic activities are present in *E. coli*. Therefore, the C_4_ sugar can be converted back to GA as soon as the aldehyde concentration decreases and no carbon is lost. In addition, the FsaA enzyme has a comparatively high Km of 62.8 mM for the acceptor GA molecule during threose formation, when compared to the Km for GA3P as acceptor molecule (0.56 mM, Sánchez-Moreno et al., 2012). Thus, threose formation can largely be prevented by adjusting an appropriate GA production rate avoiding accumulation of GA required for threose formation in the cells.

Carbon flux through the Ara5P-dependent GAA pathway was higher when the Xylu5P-specific Ca-Pkt was used when compared to the use of Ba-Pkt, which is frequently used in metabolic engineering endeavors relying on Pkt activity (Bogorad et al., 2013; Krüsemann et al., 2018; Lin et al., 2018; Wang et al., 2019). The observed 22.5% decrease of GA-derived carbon in MVA when expressing the Ba-Pkt enzyme can most likely be explained by the facts that the Ba-Pkt has a higher activity on F6P than Ca-Pkt (Bergman et al., 2016), and that F6P contains much less labelled carbon than Xylu5P (F6P: ∼10%, Xylu5P in pentose 5P pool: ∼80%, see Figure 5). In any case, the result identifies Ca-Pkt as the preferred enzyme. Furthermore, we made the observation that overexpression of the Pta enzyme had a positive impact on MVA (and therefore AcCoA) production through the synthetic pathway. This finding was rather unexpected given that Pta activity is commonly considered to be present in excess (Campos-Bermudez et al., 2010). Also, the Pkt reaction is highly irreversible (Δ_r_G'° = -58.8 kJ mol^−1^, Noor et al., 2014), which makes it very unlikely that a decrease of AcP concentration has significantly impacted the thermodynamic driving force of this reaction. While we cannot provide a conclusive explanation for the positive effects observed upon overexpressing Pta, the result supports our approach to study the function of the metabolic pathway based on an AcCoA-derived product, as this observation would not have been possible if we had only investigated the labelling pattern in acetate.

Pathway performance was extremely poor when EG was used as a starting substrate but strongly improved when EG was replaced by the product of the initial oxidation step GA. Since insufficient expression of the EG dehydrogenase enzyme could be ruled out by enzymatic measurements, this identifies the highly unfavorable thermodynamics of the NAD-dependent oxidation of EG as a major factor hampering pathway function. Due to the very high standard Gibbs energy of the EG dehydrogenase reaction (Δ_r_G'° = 23.7 kJ mol^−1^), the downstream pathway enzyme must have a very high substrate affinity for GA to enable carbon flux. For instance, the GA dehydrogenase AldA is part of the natural EG assimilation pathway in which NAD-dependent EG dehydrogenase is employed (Baldomà and Aguilar, 1987). The AldA enzyme has a very low Km of 0.14–0.38 mM for GA, thus, permitting growth of the cells on EG as sole carbon source (Baldomà and Aguilar, 1987; Rodríguez-Zavala et al., 2006). From the absence of EG assimilation through the Ara5P-dependent pathway it can be concluded that the GA affinity of the FsaA enzyme is not sufficient for enabling carbon flux (unfortunately, the Km of FsaA for GA was not reported for the Ara5P-forming reaction (Garrabou et al., 2009)). One possibility to improve EG assimilation *via* the synthetic route, therefore, is to identify or to construct an alternative Ara5P aldolase enzyme with extremely high affinity for GA. However, it appears much easier to bypass this problem by using an irreversible EG dehydrogenase in the first step of the pathway. Notably, *Pseudomonas putida* employs the irreversible pyrroloquinoline quinone-dependent PedE and PedH enzymes to oxidize EG to GA (Δ_r_G'° = -42.4 kJ mol^−1^, (Mückschel et al., 2012; Noor et al., 2014). It was previously demonstrated that *P. putida* could be used for converting EG into biomass (Mückschel et al., 2012; Li et al., 2019; Borzyskowski et al., 2022) or value-added products such as polyhydroxyalkanoates (PHA, 0.06 g_PHA_ g_EG_^−1^, Franden et al., 2018). Thus, operating the synthetic Ara5P-GAA pathway in this organism appears to be a highly interesting option for improving the yield of AcCoA-derived PHA.

Even when GA is provided as the substrate, the pathway does not produce significant amounts of MVA when no co-substrate is present. This argues for an imbalance between the recycling of the GA acceptor molecule GA3P through the synthetic pathway and the use of this metabolite in other metabolic pathways. This imbalance may be alleviated by adaptive laboratory evolution, which could be performed by extended cultivation of the cells on EG at continuously decreasing supply of the co-substrate (as applied for methanol assimilation by Keller et al., 2022). However, given the complexity of this metabolic system, it appears extremely challenging to achieve a situation where growth and product formation using EG as the sole carbon source become feasible. Therefore, it is likely that operation of the Ara5P-dependent GAA pathway will always require a co-substrate to replenish GA3P. However, the contribution of this co-substrate to the overall carbon consumption could be greatly reduced.

We could show that the Ara5P-dependent GAA pathway produced 16% of MVA (and AcCoA) when glycerol and GA are used as substrates. This result showed that the synthetic pathway can make a significant contribution to the biosynthesis of a value-added compound. While we consider this result very encouraging, it is clear that further improvements of the pathway are needed to make it relevant for use in industrial producer strains. Increasing and better balancing the activities of the pathway enzymes and chromosomal integration of the coding genes to alleviate the metabolic burden on the producer strain certainly bear considerable potential to improve pathway performance. However, we consider the use of an irreversible EG dehydrogenase, a reduced need for co-substrates, and the clarification of the role of Pta in the conversion of AcP to AcCoA as the most important factors in this regard.

## Data Availability

The raw data supporting the conclusions of this article will be made available by the authors, without undue reservation.
