# Supplementary material for: In vivo implementation of a synthetic metabolic pathway for the carbon-conserving conversion of glycolaldehyde to acetyl-CoA
Source: Front Bioeng Biotechnol. 2023 Feb 9;11:1125544. doi: 10.3389/fbioe.2023.1125544 (PMC9947464; doi:10.3389/fbioe.2023.1125544)
Supplement: Supplementary file 1 [file DataSheet1.docx]

Supplementary Material

In vivo implementation of a synthetic metabolic pathway for the carbon-conserving conversion of glycolaldehyde to acetyl-CoA

**Nils Wagner^1^, Frederik Bade^1^, Elly Straube^1^, Kenny Rabe^1^, Cláudio J.R. Frazão^1^, Thomas Walther^1*^**

^1^TU Dresden, Institute of Natural Materials Technology, Bergstraße 120, 01062 Dresden, Germany

*** Correspondence:**Thomas Walther
Email: [thomas_walther@tu-dresden.de](mailto:thomas_walther@tu-dresden.de)

**Contents Summary:**

Materials and Methods

Table S1 – Plasmids used in this work with indication of primers used for construction.

Table S2 – Primer used in this work.

Table S3 – Specific activities of purifed enzymes on their natural substrates.

Table S4 – Analyzed metabolites and their corresponding monoisotopic ion mass.

Figure S1 – Thermodynamical analysis of the proposed Ara5P-dependent GAA pathway.

Figure S2 – Growth and acetate formation during cultivation of control strain EG0 bearing an empty plasmid (pZA23_MCS) in the presence (+EG) and absence (-EG) of 500 mM ethylene glycol (EG).

Figure S3 ­– Growth and acetate formation during cultivation of strains EG1 (A), EG2 (B), EG3 (C), EG5(D), EG6 (E) and EG7 (F) in the presence (+EG) and absence (-EG) of 500 mM 13C2-ethylene glycol (EG).

Figure S4 – Concentration courses of consumed substrates and related side products detected in the supernatant of strain GA1 during 24 h of incubation

Figure S5 – Growth and production profile of EG8 cultivated in presence of ^13^C_2_ ethylene glycol (EG)

Material and methods
Enzymes used for the *in vitro* demonstration of the synthetic pathway were tested in advance for functional expression on the respective natural substrates. pET-28a(+) plasmids with the corresponding genes were expressed in *E. coli* BL21(DE3) cells and purified as described in material and methods of the main manuscript. Enzyme activity for used fructose 6-phosphate aldolase (FsaA), arabinose 5-phosphate isomerase (KdsD), ribulose-phosphate 3-epimerase (Rpe) and phosphoketolase from *C. acetobutylicum* (Ca-Pkt) were quantified. All assays were performed in 96-well microtiter plates at 30 °C.

FsaA enzyme assay
Functional expression of purified FsaA was measured on fructose 6-phosphate (F6P) using a protocol adapted from Schürmann and Sprenger, 2001 with the two commercial (Sigma-Aldrich, USA) helper enzymes triose phosphate isomerase (TPI) and glycerol 3-phosphate dehydrogenase (GAPDH) isolated from rabbit muscle. In the first step, F6P is converted by FsaA to dihydroxyacetone (DHA) and glyceraldehyde-3-phosphate (GA3P). This is followed by the conversion of GA3P to dihydroxyacetone phosphate (DHAP) by the TPI. In the last step, DHAP is reduced to glycerol-3-phosphate (G3P) by GPDh and at the same time NADH is oxidized to NAD. Decrease of NADH was followed at 340 nm (ɛ_340 nm_= 6.22 mM cm^-1^). The reaction mix contained the purified FsaA protein solution, 50 mM HEPES buffer (pH 7.5), 40 U of TPI, 40 U of GAPDH, 0.5 mM NADH and 25 mM F6P.

Rpe enzyme assay
Rpe activity was measured on ribulose 5-phosphate (Ribu5P) coupled with purified phosphoketolase (Ca-pkt) enzyme solution and the two commercial (Sigma-Aldrich, USA) helper enzymes TPI and GAPDH isolated from rabbit muscle. Ribu5P is converted into xylulose 5-phosphate (Xylu5P), which is cleaved by the Ca-Pkt into acetyl-phosphate (AcP) and GA3P. Catalyzed by TPI DHA is formed from GA3P and further reduced to G3P under oxidation of NADH to NAD. Decrease of NADH was followed at 340 nm (ɛ_340 nm_= 6.22 mM cm^-1^). Assay mix contained 50 mM HEPES buffer (pH 7.5), 33 mM KH_2_PO_4_, 1.25 mM Thiamine pyrophosphate (TPP), 5 mM MgCl_2_, 5 mM Ribu5P, 10 U TPI, 10 U GAPDH, 100 µg mL^-1^ Ca-Pkt.

KdsD enzyme assay
Functional expression of purified KdsD was investigated by a cysteine-carbazole colorimetric assay as described by (Meredith and Woodard, 2003). Activity was measured on 2.5 mM arabinose 5-phosphate (Ara5P) and formation of Ribu5P was quantified. Enzyme solutions were diluted in Tris HCl buffer (50 mM) of pH 7.5. Ribu5P standards were used for calibration.

Pkt enzyme assay
Ca-pkt activity was measured using F6P as substrate. The formation of AcP was detected using a colorimetric ferric-acetyl-hydroxamate assay (Lipmann and Tuttle, 1945; Racker, 1962). The reaction mix (80 µL) consists purified Ca-pkt enzyme solution, 40 mM HEPES buffer (pH 7.5), 20 mM KH_2_PO_4_, 17.25 mM NaF, 3.75 mM MgCl_2,_ 1.4 mM L-cystein, 6 mM iodoacetate, 0.75 mM TPP and 25 mM F6P. After 30 min of incubation, the reaction was stopped by the addition of 60 µL 2 M hydroxylamine solution (adjusted to pH 6.5) and placed at room temperature. 10 min later, 40 µL each of (15 % w/v) trichloroacetic acid, 4 M HCl and (5 % w/v in 0.1 M HCl) FeCl_3_ solution was added to generate ferric-acetyl-hydroxamate complexes, which were detected at a wavelength of 505 nm. For the calibration the same reaction mix containing lithium AcP standards (0 – 14 mM) instead of F6P and enzyme solution were carried.

Table S1. Plasmids used in this work with indication of primers used for construction.

| - **Name** | - **Relevant characteristics** | - **Primer used for construction** | - **Reference/ origin** | |
| --- | --- | --- | --- | --- |
| - pET-28(a)+ | *ori f1*, Kan^R^, T7 promoter |  | - Novagen™ | |
| - pCP20 | ori pSC101, Amp^R^, Cm^R^,  plasmid expressing Flp recombinase to remove Kan cassette |  | - (Cherepanov and Wackernagel, 1995) | |
| - pMEV-7 | - *ori colE1*, Amp^R^, P_LlacO_ promoter, *lacI*^q^,  carrying *atoB* from *E. coli*, *mvaS* and *mvaA* from *L. casei* |  | - (Xiong et al., 2014) | |
| - pKD4 | - Amp^R^ |  | - (Datsenko and Wanner, 2000) |  |
| - pKD46 | - Amp^R^ |  | - (Datsenko and Wanner, 2000) |  |
| - pZA23 | - ori *p15A*, Kan^R^, P_A1lacO-1_ promoter |  | - (Lutz and Bujard, 1997) |  |
| - pZS13 | - *ori pSC101*, Amp^R^, P_A1lacO-1_ Promotor |  | - (Lutz and Bujard, 1997) |  |
| - pZS33 | - *ori pSC101*, Cm^R^, P_A1lacO-1_ Promotor |  | - (Lutz and Bujard, 1997) |  |
| - pET28_Ca-pkt | - pET-28(a)+ carrying *xfp* gene from *C. acetobutylicum* |  | - This study |  |
| - pET28_fsaA | - pET-28(a)+ carrying *fsaA* |  | - This study |  |
| - pET28_kdsD | - pET-28(a)+ carrying *kdsD* |  | - This study |  |
| - pET28_rpe | - pET-28(a)+ carrying *rpe* |  | - This study |  |
| - pZA23_fsaA_Ca-pkt | - pZA23 carrying *fsaA* and *xfp* gene from *C. acetobutylicum* | - TW366, TW367, TW555, TW236, TW237, TW371 | - This study |  |
| - pZA23_talB_Ca-pkt | - pZA23 carrying *talB* and *xfp* gene from *C. acetobutylicum* | - TW366, TW367, TW2021, TW2022, TW2063, TW371 | - This study |  |
| - pZA23_talB^F178Y^_Ca-pkt | - pZA23 carrying *talB^F178Y^* and *xfp* gene from *C. acetobutylicum* | - TW366, TW367, TW2021, TW2026, TW2022, TW2025, TW2063, TW371 | - This study |  |
| - pZA23_fsaA_Ba-pkt | - pZA23 carrying *fsaA* and *xfp* gene from *B. adolescentis* | - TW366, TW367, TW555, TW236, TW1347, T1348 | - This study |  |
| - pZA23_fucO^Mut^ | - pZA23 carrying *fucO^I6L L7V^* | - TW366, TW367, TW368, TW369 | - This study |  |
| - pZA23_fucO^Mut^_Ca-pkt | - pZA23 carrying *fucO^I6L L7V^* and *xfp* gene from *C. acetobutylicum* | - TW366, TW367, TW368, TW203, TW204, TW371 | - This study |  |
| - pZA23_fucO^Mut^_fsaA_Ca-pkt | - pZA23 carrying *fucO^I6L L7V^*, *fsaA* and *xfp* gene from  *C. acetobutylicum* | - TW366, TW367, TW368, TW200, TW201, TW236, TW237, TW371 | - This study |  |
| - pZS13_kdsD | - pZS13 carrying *kdsD* | - TW293, TW294, TW295, TW2023 | - This study |  |
| - pZS13_rpe | - pZA13 carrying *rpe* | - TW293, TW294, TW297, TW2024 | - This study |  |
| - pZS13_kdsD_rpe | - pZS13 carrying *kdsD* and *rpe* | - TW293, TW294, TW295, TW296, TW297, TW298 | - This study |  |
| - pZS33_kdsD_rpe | - pZS33 carrying *kdsD* and *rpe* | - TW293, TW294, TW295, TW296, TW297, TW298 | - This study |  |
| - pZS13_kdsD_rpe_pta | - pZS33 carrying *kdsD*, *rpe and pta* | - TW293, TW294, TW295, TW296, TW297, TW1339, TW1340, TW1341 | - This study |  |

Table S2. Primer used in this work.

| **Ref.** | **Sequence** | | **Restriction enzymes** |
| --- | --- | --- | --- |
| **Primers for cloning into pET-28(a)+ derived expression vectors** | | | |
| - *Ca-Pkt (Q97JE3)* | AGATATGCTAGCATGCAAAGTATAATAGGAAAACATAAGG AGATATGGATCCTTATACATGCCACTGCCAATTAG | NdeI  BamHI | |
| - *FsaA (P78055)* | AGATATCATATGGAACTGTATCTGGATACTTCAGAC AGATATGAATTCTTAAATCGACGTTCTGCCAAACGC | NdeI  EcoRI | |
| - *KdsD (P45395)* | AGATATGCTAGCATGTCGCACGTAGAGTTACAACC AGATATGAATTCTTACACTACGCCTGCACGCAG | NdeI  EcoRI | |
| - *Rpe (P0AG07)* | AGATATCATATGAAACAGTATTTGATTGCCCCCTC AGATATGAATTCTTATTCATGACTTACCTTTGCCAGT | NdeI  EcoRI | |
| **Primers for construction of pZS13, pZS33 and pZA23-derived expression vectors by** | | | **Application** |
| - TW200 | - TAAAAACTCTCCTTATCCTTGTTGGTCGCTGGTACAAAGCTTTTACCAGGCGGTATGGTAA | | - Amplification of *fucO^Mut^* (rv) |
| - TW201 | TGTACCAGCGACCAACAAGGATAAGGAGAGTTTTTAATGGAACTGTATCTGGATAC | | - Amplification of *fsaA* (fw) |
| - TW203 | AAATGACCTCCTTAACTATCAAATTTTGCCCAGTACGCAAGCTTTTACCAGGCGGTATGGTAA | | - Amplification of Ca-*xfp* (fw) |
| - TW204 | GCGTACTGGGCAAAATTTGATAGTTAAGGAGGTCATTTATGCAAAGTATAATAGGAAAAC | | - Amplification of Ca-*xfp* (rv) |
| - TW236 | TAAAAACCTCCTTACTATTCCTCTTGTGATTATCTGGGGTCGATCAGTTAAATCGACGTTCTGCC | | - Amplification of *fsaA* (rv) |
| - TW237 | AAGAGGAATAGTAAGGAGGTTTTTAATGCAAAGTATAATAGGAAAAC | | - Amplification of Ca-*xfp* (fw) |
| - TW293 | TGACTCTAGAGGCATCAAATA | | - Amplification pZS13/pZS33 backbone (fw) |
| - TW294 | GGTACCTTTCTCCTCTTTAAT | | - Amplification pZS13/pZS33 backbone + pZ-RBS (rv) |
| - TW295 | ATTCATTAAAGAGGAGAAAGGTACCATGTCGCACGTAGAGTTACAACCG | | - Amplification of *kdsD* (fw) |
| - TW296 | AAAAAACCTCCTTAGTAAATATATTTCTAATATTCGCAGGCGTAGATTACACTACGCCTGCACGCA | | - Amplification of *kdsD* (rv) |
| - TW297 | AATATATTTACTAAGGAGGTTTTTTATGAAACAGTATTTGATTGCCC | | - Amplification of *rpe* (fw) |
| - TW298 | GTTTTATTTGATGCCTCTAGAGTCATTATTCATGACTTACCTTTGCC | | - Amplification of *rpe* (rv) |
| - TW366 | GGATCCCATGGTACGCGTGC | | - Amplification pZA23 backbone (fw) |
| - TW367 | GGTATATCTCCTTCTTAAAGTTAAACTGAATTCTGTGTGAAATTGTTATCCGC | | - Amplification pZA23 backbone + pET28-RBS (rv) |
| - TW368 | GTTTAACTTTAAGAAGGAGATATACCATGGCTAACAGAATGCTTGT | | - Amplification of *fucO^Mut^* (fw) |
| - TW369 | - GCACGCGTACCATGGGATCCTTACCAGGCGGTATGGTAAA | | - Amplification of *fucO^Mut^* (rv) |
| - TW371 | - GCACGCGTACCATGGGATCCTTATACATGCCACTGCCAAT | | - Amplification of Ca-*xfp* (rv) |
| - TW555 | - GTTTAACTTTAAGAAGGAGATATACCATGGAACTGTATCTGGATACTTC | | - Amplification of *fsaA* (fw) |
| - TW1339 | - AGATAGAGGTAGTTTTGGATTTATTCATGACTTACCTTTGCCAG | | - Amplification of *rpe* (rv) |
| - TW1340 | - ATTTGATGCCTCTAGAGTCATTACTGCTGCTGTGCAGAC | | - Amplification of *pta* (rv) |
| - TW1341 | - ATCCAAAACTACCTCTATCTTAAGGCCTAAATGTCCCGTATTATTATGCTGATC | | - Amplification of *pta* (fw) |
| - TW1347 | - GAATAGTAAGGAGGTTTTTAATGACGAGTCCTGTTATTGGCA | | - Amplification of Ba-*xfp* (fw) |
| - TW1348 | - GCACGCGTACCATGGGATCCTTACTCGTTATCGCCAGCGG | | - Amplification of Ba-*xfp* (rv) |
| - TW2021 | - AGAAGGAGATATACCATGACGGACAAATTGACCTCC | | - Amplification of *talB* (fw) |
| - TW2022 | - TCTGGGGTCGATCAGTTACAGCAGATCGCCGATCA | | - Amplification of *talB* (rv) |
| - TW2023 | - TGCCTCTAGAGTCATTACACTACGCCTGCAC | | - Amplification *kdsD* (rv) |
| - TW2024 | - GAGAAAGGTACCATGAAACAGTATTTGATTGCCC | | - Amplification *rpe* (fw) |
| - TW2025 | - CGCCGTATGTTGGCCGTATTCTTGACTGGTA | | - Amplification of *talB_F178Y* (fw) |
| - TW2026 | - CGGCCAACATACGGCGAGATCAGGAACACG | | - Amplification of *talB_F178Y* (rv) |
| - TW2063 | - CTGATCGACCCCAGATAATCAC | | - Amplification of Ca-*xfp* (fw) |
| - **Primers for chromosomal integration of synthetic ProD-promoter** | | | |
| - TW1553 | - GATGTTGTACTGGTTATCGCCAATACTCGTTGAATAACTGGAAACGCATTGTGTAGGCTGGAGCTGCTTC | | - Amplification of Kan^R^-ProD cassette for integration at *kdsD* locus (fw) |
| - TW1554 | - TTACCTGCTTGCTGAAAGTCAAAACCCGGTTGTAACTCTACGTGCGACATATAATACCTCCTAAAGTTAAACAAAATTATTTGTAG | | - Amplification of Kan^R^-ProD cassette for integration at *kdsD* locus (rv) |
| - TW1555 | - CGGGCAAAATCAGCCGACAGAATTGAGGGGGCAATCAAATACTGTTTCATATAATACCTCCTAAAGTTAAACAAAATTATTTGTAG | | - Amplification of Kan^R^-ProD cassette for integration at *rpe* locus (fw) |
| - TW1556 | - AAACCAGGAGTCGTTTCACCCGCGAAAAAATAATTCTCAAGGAGAAGCGGGTGTAGGCTGGAGCTGCTTC | | - Amplification of Kan^R^-ProD cassette for integration at *rpe* locus (rv) |
| **Primers for verification of chromosomal manipulations** | | | |
| - TW216 | - TGCGGCAATTTGATTG | | - verify *yqhD* deletion (fw) |
| - TW217 | - CATTACTTGCTTGCCAGACG | | - verify *yqhD* deletion (rv) |
| - TW218 | - ACGGGCATGACTCCTG | | - verify *aldA* deletion (fw) |
| - TW219 | - GGCGATTTACAACTGGTGAA | | - verify *aldA* deletion (rv) |
| - TW596 | - CCAATCAGCAACGACTGTTT | | - verify *lacI^q^-Spc^R^* integration (fw) |
| - TW597 | - CGTACATTTGTACGGCTCC | | - verify *lacI^q^-Spc^R^* integration (rv) |
| - TW1595 | - GTTGACGATGGGTCTGAC | | - verify ProD integration in front of *kdsD* (fw) |
| - TW1596 | - TAACAGGTCGCAAGCGAC | | - verify ProD integration in front of *kdsD* (rv) |
| - TW1597 | - GTCGACCAGCGTACCATCAAG | | - verify ProD integration in front of *rpe* (fw) |
| - TW1598 | - CGGCGTATCACACAAAC | | - verify ProD integration in front of *rpe* (rv) |

Table S3. Specific activities of purified enzymes on their natural substrates. Enzymatic activities were determined at 30 °C (n≥3). D-fructose 6-phosphate, F6P; D-arabinose 5‑phosphate, Ara5P; D-ribulose 5-phosphate, Ribu5P

| **Enzyme** | **FsaA** | **KdsD** | **Rpe** | **Ca-Pkt** |
| --- | --- | --- | --- | --- |
| **Substrate** | F6P | Ara5P | Ribu5P | F6P |
| **Activity [U mg^-1^]** | 1.09 ± 0.15 | 0.52 ± 0.04 | 1.11 ± 0.25 | 1.47 ± 0.28 |

Table S4. Analyzed metabolites and their corresponding monoisotopic ion mass. All LC/MS measurements were performed using negative ion mode ([M-H]-) except for the extracellular metabolite acetate, which was measured using positive ion mode ([M+H]+).

| Metabolite | Chemical formula | Monoisotopic ion [M-H]- |
| --- | --- | --- |
| Acetate | C_2_O_2_H_4_ | 61.02841 [M+H]+ |
| Acetyl phosphate | C_2_H_5_O_5_P | 138.98018 |
| Adenosine monophosphate | C_10_H_14_N_5_O_7_P | 346.05581 |
| Aspartate | C_4_H_7_NO_4_ | 132.03023 |
| Cytidine monophosphate | C_9_H_14_N_3_O_8_P | 322.04457 |
| Fructose 6-phosphate/ Glucose 6-phosphate | C_6_H_13_O_9_P | 259.02244 |
| Glyceraldehyde 3-phosphate/ DHAP | C_3_H_7_O_6_P | 168.99075 |
| Glutamate | C_5_H_9_NO_4_ | 146.04588 |
| Leucine | C_6_H_13_NO_2_ | 130.08735 |
| Malate | C_4_H_6_O_5_ | 133.01425 |
| Mevalonate | C_6_O_4_H_12_ | 147.06628 |
| 2-Oxoglutarate | C_5_H_6_O_5_ | 145.01425 |
| Pentose 5-phosphates | C_5_H_11_O_8_P | 229.01188 |
| Phospo*enol*pyruvate | C_3_H_5_O_6_P | 166.97510 |
| Pyruvate | C_3_H_4_O_3_ | 87.00877 |
| Sedoheptulose 7-phosphate | C_7_H_15_O_10_P | 289.03301 |
| Tryptophan | C_11_H_12_N_2_O_2_ | 203.08260 |


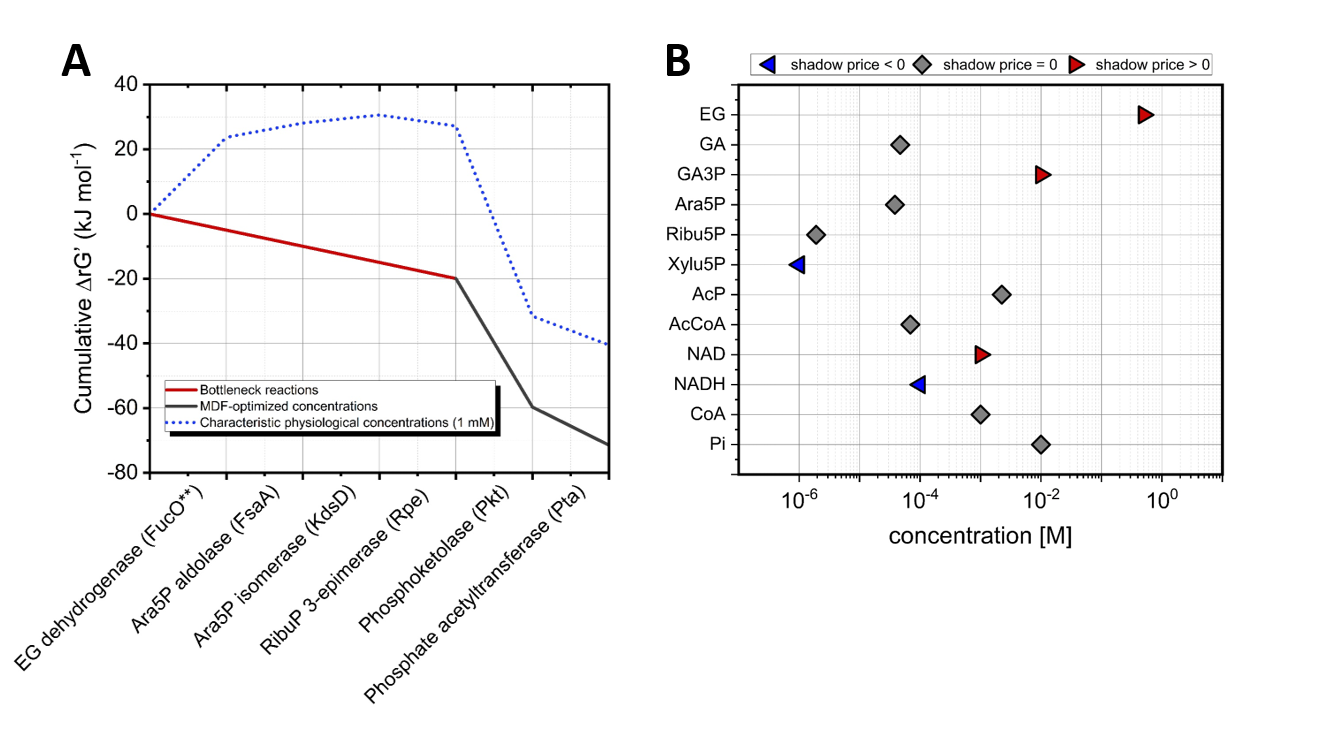


Figure S1 – Max-min Driving Force (MDF) analysis (Noor et al., 2014) of the proposed Ara5P-dependent GAA pathway. (A) Energetic profile of the reaction sequence starting with the substrate ethylene glycol (fixed to 500 mM). Blue line shows the cumulative Gibbs energy in respect to fixed metabolite concentrations of 1 mM each (∆r G’^m^, pH 7). Gray line corresponds to ∆r G’ values of pathway reactions after MDF optimization, whereas the red line represents predicted bottleneck reactions. (B) Optimal metabolite concentration after MDF optimization within physiological range (1µM – 10 mM). Cofactors NAD, NADH and coenzyme A (CoA) have fixed concentrations (Noor et al., 2014). Components that limited the driving force according to the MDF analysis are shown in red (positive shadow price).

Calculations shown in Figure S1 indicating that the initial NAD-dependent ethylene glycol oxidation is under non-optimized conditions a thermodynamic hurdle (ΔrG'° = 23.7 kJ mol^-1^, FucO^Mut^/FucO**). Mainly responsible for the thermodynamic driving force via the Ara5P-dependent GAA metabolic pathway is the reaction catalyzed by the heterologous Pkt (-58.8 kJ mol^-1^). According to the ΔrG'° of the reversible reaction from Ara5P to Ribu5P (2.5 kJ mol^-1^, KdsD) and further to Xylu5P (-3.4 kJ mol^‑1^, Rpe) a quasi-equilibrium is expected for these compounds.


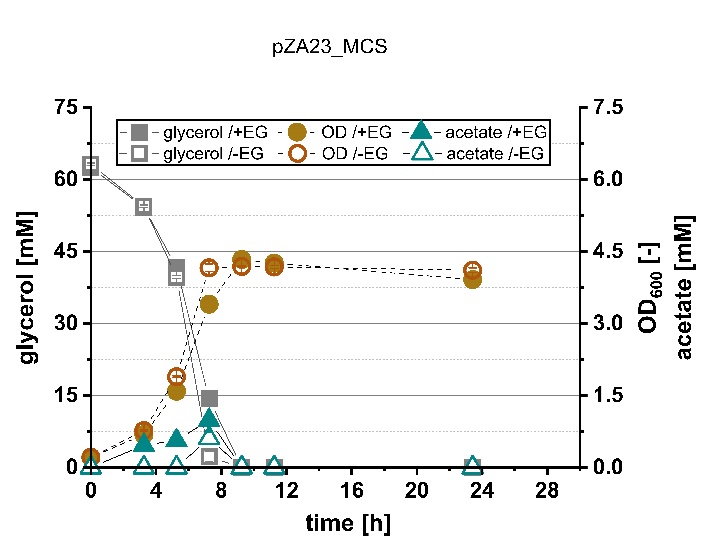


Figure S2. Growth and acetate formation during cultivation of control strain EG0 bearing an empty plasmid (*E. coli* Δ*yqhD* Δ*aldA lacIq* + pZA23_MCS) in the presence (+EG) and absence (-EG) of 500 mM ethylene glycol (EG). Cells were cultivated in minimal medium (M9) supplemented with 55 mM glycerol. Error bars indicate standard error of the mean (n=2).


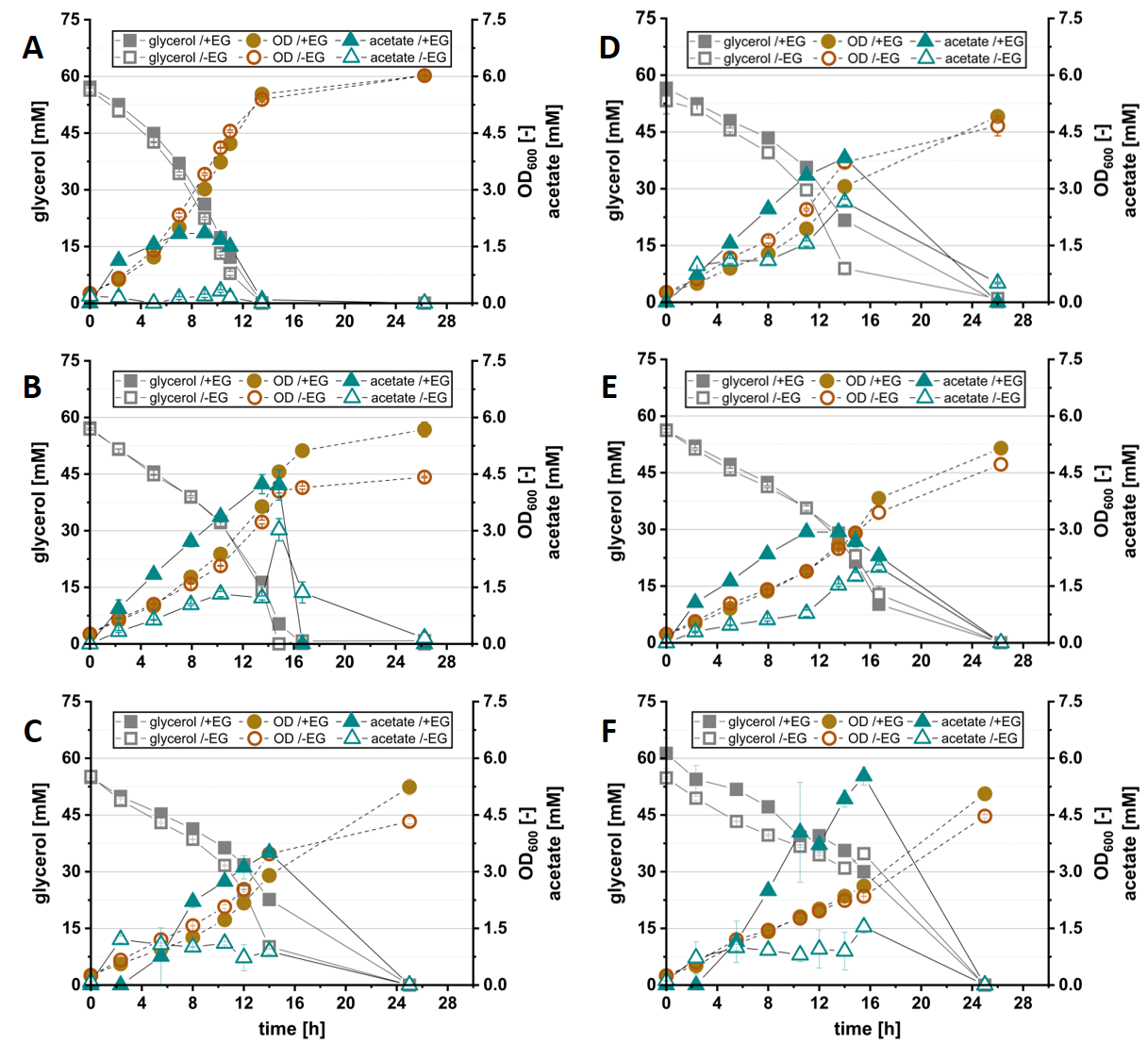


Figure S3. Growth and acetate formation during cultivation of strains EG1 (A), EG2 (B), EG3 (C), EG5 (D), EG6 (E) and EG7 (F) in the presence (+EG) and absence (-EG) of 500 mM ^13^C_2_-ethylene glycol (EG). Cells were cultivated in minimal medium (M9) supplemented with 55 mM glycerol. Protein expression was induced by addition of 1 mM IPTG at an optical density at 600 nm (OD_600_) of ~ 0.6. Error bars indicate standard error of the mean (n=2).


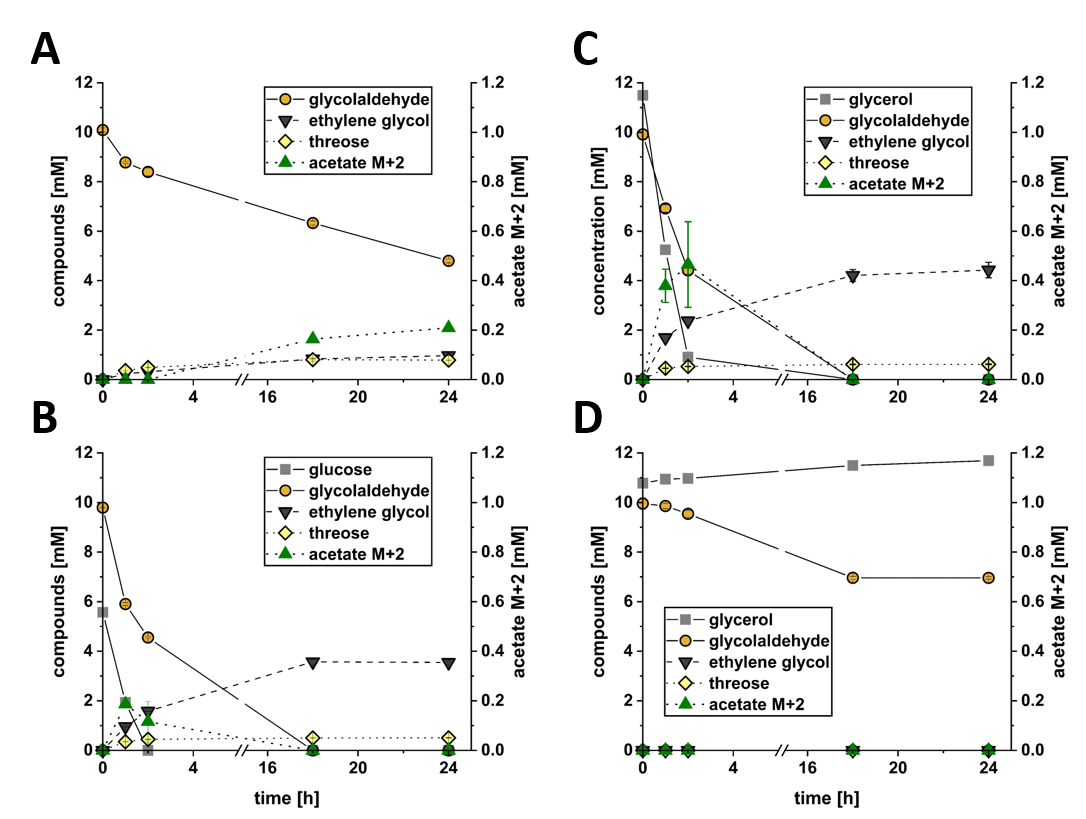


Figure S4. Concentration courses of consumed substrates and related side products detected in the supernatant of strain GA1 during 24 h of incubation. Strain GA1 (*E. coli* Δ*yqhD* Δ*aldA lacIq* + pZA23_fsaA_Ca-pkt + pZS33_kdsD_rpe + pMEV-7) was incubated in minimal M9 medium supplemented with 1 mM IPTG and 10 mM ^13^C_2_-GA at 30 °C (A) without addition of a co-substrate or in presence of either (B) 5 mM glucose or (C) 10 mM glycerol. Control experiment (D) was conducted in absence of cells but with addition of glycerol (10 mM). Error bars indicate standard error of the mean (n=2).


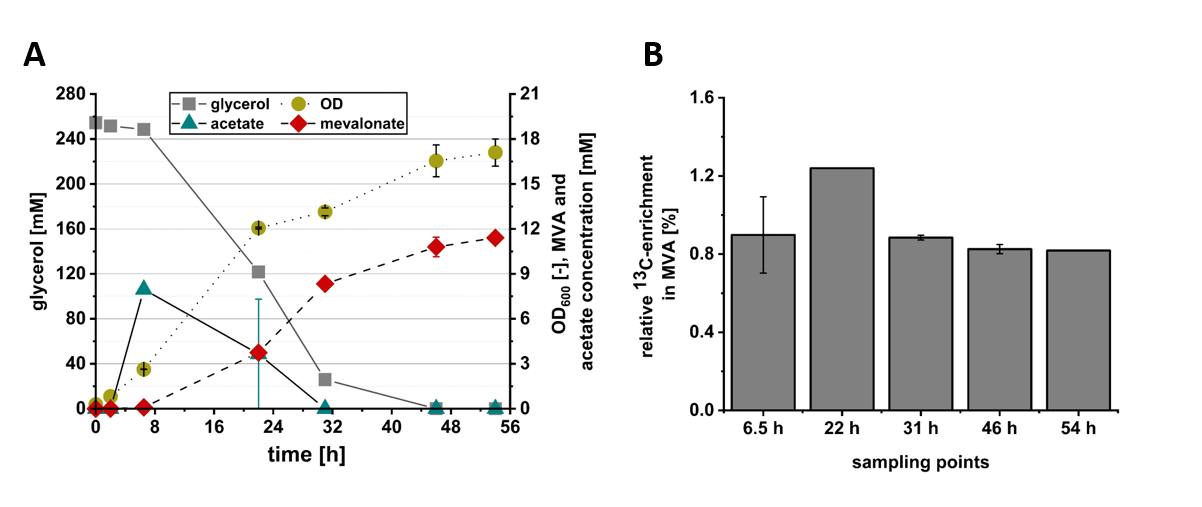


Figure S5. Growth and production profile of EG8 cultivated in presence of ^13^C_2_ ethylene glycol (EG). (A) Growth, glycerol uptake and product formation kinetics of strain EG8 (*E. coli* Δ*yqhD* Δ*aldA lacIq* + pZA23_fucO^Mut^_fsaA_Ca-pkt + pZS33_kdsD_rpe_pta + pMEV-7). (B) Relative enrichment of ^13^C-carbon found in the product MVA for the specific sampling points. Values were calculated by dividing amount of labelled C-atoms by total amount of C-atoms. Strain EG8 was cultivated on mineral medium containing 250 mM glycerol, 5 g L^-1^ yeast extract, 100 mM CaCO_3_ and 500 mM ^13^C_2_-EG. Protein expression was induced after 2 h of cultivation by addition of 1mM IPTG. Error bars indicate standard error of the mean (n=2).

# Supplementary references

Cherepanov, P. P., and Wackernagel, W. (1995). Gene disruption in Escherichia coli: TcR and KmR cassettes with the option of Flp-catalyzed excision of the antibiotic-resistance determinant. *Gene* 158, 9–14. doi: 10.1016/0378-1119(95)00193-A.

Datsenko, K. A., and Wanner, B. L. (2000). One-step inactivation of chromosomal genes in Escherichia coli K-12 using PCR products. *Proc. Natl. Acad. Sci. U. S. A.* 97, 6640–6645. doi: 10.1073/pnas.120163297.

Lipmann, F., and Tuttle, L. Constanc. (1945). A SPECIFIC MICROMETHOD FOR THE DETERMINATION OF ACYL PHOSPHATES. *J. Biol. Chem.* 159, 21–28. doi: 10.1016/S0021-9258(19)51298-4.

Lutz, R., and Bujard, H. (1997). Independent and Tight Regulation of Transcriptional Units in Escherichia Coli Via the LacR/O, the TetR/O and AraC/I1-I2 Regulatory Elements. *Nucleic Acids Res.* 25, 1203–1210. doi: 10.1093/nar/25.6.1203.

Meredith, T. C., and Woodard, R. W. (2003). Escherichia coli YrbH Is a D-Arabinose 5-Phosphate Isomerase*. *J. Biol. Chem.* 278, 32771–32777. doi: 10.1074/jbc.M303661200.

Noor, E., Bar-Even, A., Flamholz, A., Reznik, E., Liebermeister, W., and Milo, R. (2014). Pathway Thermodynamics Highlights Kinetic Obstacles in Central Metabolism. *PLOS Comput. Biol.* 10, e1003483. doi: 10.1371/journal.pcbi.1003483.

Racker, E. (1962). “[29d] Fructose-6-phosphate phosphoketolase from Acetobacter xylinum: F-6-P+Pi→Acetyl-P+E-4-P+H2O,” in *Methods in Enzymology* (Academic Press), 276–280. doi: 10.1016/S0076-6879(62)05219-2.

Schürmann, M., and Sprenger, G. A. (2001). Fructose-6-phosphate Aldolase Is a Novel Class I Aldolase from Escherichia coli and Is Related to a Novel Group of Bacterial Transaldolases*. *J. Biol. Chem.* 276, 11055–11061. doi: 10.1074/jbc.M008061200.

Xiong, M., Schneiderman, D. K., Bates, F. S., Hillmyer, M. A., and Zhang, K. (2014). Scalable production of mechanically tunable block polymers from sugar. *Proc. Natl. Acad. Sci.* 111, 8357–8362. doi: 10.1073/pnas.1404596111.
